# Discovery of Guanfacine as a Novel TAAR1 Agonist: A Combination Strategy through Molecular Modeling Studies and Biological Assays

**DOI:** 10.3390/ph16111632

**Published:** 2023-11-20

**Authors:** Elena Cichero, Valeria Francesconi, Beatrice Casini, Monica Casale, Evgeny Kanov, Andrey S. Gerasimov, Ilya Sukhanov, Artem Savchenko, Stefano Espinoza, Raul R. Gainetdinov, Michele Tonelli

**Affiliations:** 1 Department of Pharmacy, Section of Medicinal Chemistry, School of Medical and Pharmaceutical Sciences, University of Genoa, 16132 Genoa, Italy; elena.cichero@unige.it (E.C.); valeria.francesconi@edu.unige.it (V.F.); beatrice.casini.bc@gmail.com (B.C.); 2 Section of Chemistry and Food and Pharmaceutical Technologies, University of Genoa, 16148 Genoa, Italy; monica.casale@unige.it; 3 Institute of Translational Biomedicine, St. Petersburg State University, 199034 St. Petersburg, Russia; e.kanov@spbu.ru (E.K.); asgerasimoff@mail.ru (A.S.G.); gainetdinov.raul@gmail.com (R.R.G.); 4 St. Petersburg University Hospital, St. Petersburg State University, 199034 St. Petersburg, Russia; 5 Valdman Institute of Pharmacology, Pavlov First St. Petersburg State Medical University, 197022 St. Petersburg, Russia; ilia.sukhanov@gmail.com (I.S.); artjom1996@yandex.ru (A.S.); 6 Department of Health Sciences and Research Center on Autoimmune and Allergic Diseases (CAAD), University of Piemonte Orientale (UPO), 28100 Novara, Italy; stefano.espinoza@uniupo.it; 7 Central RNA Laboratory, Istituto Italiano di Tecnologia (IIT), 16152 Genova, Italy

**Keywords:** AlphaFold, GPCR, docking, QSAR, guanfacine, TAAR1, α_2_-adrenoreceptor, dopamine

## Abstract

Trace amine-associated receptor 1 (TAAR1) is an attractive target for the design of innovative drugs to be applied in diverse pharmacological settings. Due to a non-negligible structural similarity with endogenous ligands, most of the agonists developed so far resulted in being affected by a low selectivity for TAAR1 with respect to other monoaminergic G protein-coupled receptors, like the adrenoreceptors. This study utilized comparative molecular docking studies and quantitative–structure activity relationship (QSAR) analyses to unveil key structural differences between TAAR1 and alpha2-adrenoreceptor (α_2_-ADR), with the aim to design novel TAAR1 agonists characterized by a higher selectivity profile and reduced off-target effects. While the presence of hydrophobic motives is encouraged towards both the two receptors, the introduction of polar/positively charged groups and the ligand conformation deeply affect the TAAR1 or α_2_-ADR putative selectivity. These computational methods allowed the identification of the α_2_A-ADR agonist guanfacine as an attractive TAAR1-targeting lead compound, demonstrating nanomolar activity in vitro. In vivo exploration of the efficacy of guanfacine showed that it is able to decrease the locomotor activity of dopamine transporter knockout (DAT-KO) rats. Therefore, guanfacine can be considered as an interesting template molecule worthy of structural optimization. The dual activity of guanfacine on both α_2_-ADR and TAAR1 signaling and the related crosstalk between the two pathways will deserve more in-depth investigation.

## 1. Introduction

Trace amine-associated receptor 1 (TAAR1) is a promising target for the development of innovative therapies for many diseases, in virtue of its wide distribution in the brain and in diverse peripheral tissues [1,2,3,4,5]. TAAR1 is responsive to a class of biogenic compounds called trace amines (TAs), such as tyramine (TYR), β-phenylethylamine (β-PEA), and 3-iodothyronamine (T1AM), whose dysregulation was correlated to the etiology of various diseases, like schizophrenia, depression, attention deficit hyperactivity disorder, substance abuse, metabolic syndrome, and Parkinson’s disease [3,6,7,8,9,10,11]. In the brain, TAAR1 proved to be an important modulator of the major monoamine (dopamine and serotonin) and glutamate signaling pathways, directing the attention of researchers on the therapeutic implications of TAAR1 ligands in neuropsychiatric disorders [3,12,13]. The pharmaceutical company Hoffmann-La Roche was an early leader in TAAR1 drug discovery and registered several patents [14] as well as published preclinical studies [15,16,17,18,19] with selective TAAR1 agonists. TAAR1 agonists now give promise to be a new generation of antipsychotic medications, as evidenced by two compounds that have entered clinical trials, SEP-363856 (ulotaront—Sunovion) and RO6889450 (ralmitaront—La Roche) [13,20,21]. While there is limited publicly available information on ralmitaront’s preclinical profile [22], ulotaront is designated as a promiscuous TAAR1 and 5-HT1A agonist [23,24] that is proving able to control the positive, negative, and cognitive symptoms of schizophrenia [25,26]. Notably, ulotaront seems to lack the class-specific adverse events of traditional D2 or 5-HT2A antipsychotics [20,27], such as the weight gain, metabolic issues, and Parkinson-like symptoms.

Since the beginning, the study of TAAR1 ligands chemotype took inspiration from the chemical scaffold of endogenous TAs, leading to the identification of several analogues often endowed with a more promising *m*TAAR1 affinity, rather than towards the *h*TAAR1 ortholog [28]. During the last years, the species-specificity issue was explored by our group based on molecular modelling studies of murine (*m*TAAR1) and human TAAR1 (*h*TAAR1) receptors. As a result, we developed some novel TAAR1 chemotypes, namely the phenyl(benzyl) biguanides and the piperazine-containing biguanides, which showed varying micromolar activity towards the two receptors [29,30]. Successively, through the combination of a pharmacophore model and a scaffold simplification strategy of the previous biguanide-based TAAR1 agonists, a new series of 1-amidino-4-phenylpiperazine derivatives was developed and provided nanomolar functional activity at *h*TAAR1 and low cytotoxicity [31]. All these molecules shared the key structural features required for a TAAR1-targeting activity, as a basic core forming a key salt-bridge with a conserved *m/h*TAAR1 D3.32 aspartic acid (namely Asp102 and Asp103, respectively) and an aromatic moiety forming π-π stacking and van der Waals interactions with a number of aromatic residues (see below) [32].

**Figure 1:**
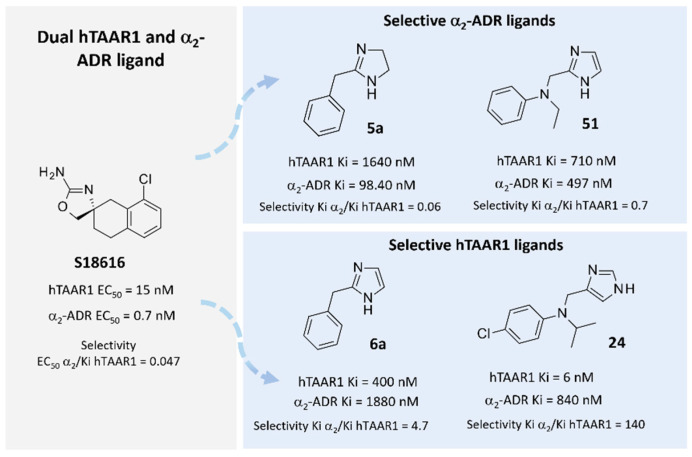
Chemical structure and biological activity of the reference dual acting TAAR1 and α_2_-ADR ligand **S18616**. Representative examples of TAAR1 or α_2_-ADR selective imidazoline and imidazole derivatives [35].

The discovery of novel TAAR1-targeting ligands also moved through numerous screening campaigns involving known dopaminergic, serotonergic, and adrenergic drugs [33]. This strategy led to several series of TAAR1 agonists, which confirmed their efficacy at the expense of selectivity over other G-protein-coupled receptors (GPCRs) [32]. In this regard, compound **S18616** [34] was reported in the literature as a potent alpha2-adrenoreceptor (α_2_-ADR) agonist and then also evaluated as a TAAR1 agonist. To pursue more selective TAAR1 or α_2_-ADR ligands, structural variations of the main **S18616** tricyclic ring was afforded, leading to different series of imidazoline, imidazole, [35] and amino oxazoline [36] derivatives (Figure 1).

Herein, we collected in silico the reference **S18616** and the previously cited dual acting compounds (**5a**-**5e**, **6a**-**6e**, **11**-**54**), exhibiting in particular the imidazoline and imidazole chemotype, for the following computational studies. The agonist selectivity profile as TAAR1 and/or α_2_-ADR ligands was then investigated based on deepening comparative molecular docking studies and quantitative–structure activity relationship (QSAR) analyses. The results pointed out the key structural differences between the two receptors, the most relevant pharmacophore features, and chemical descriptors turning in the agonist selectivity. The derived information is expected to be useful for the design of more selective TAAR1 ligands as a further prosecution of this work.

**Figure 2:**
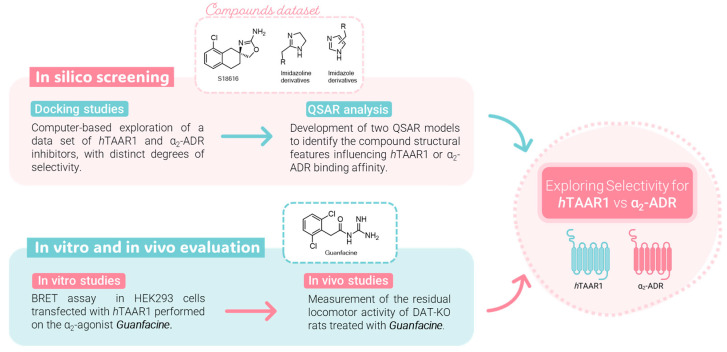
Workflow of the present study: a combination approach through molecular modeling studies and biological assays.

To preliminary assess the minimum criteria to achieve TAAR1 and/or α_2_-ADR agonism, as determined by previously mentioned computational studies, we identified guanfacine, a 2,6-dichlorophenylacetyl guanidine derivative, as a novel *m*/*h*TAAR1 agonist. A schematic representation of the whole developed study is reported in Figure 2.

Originally approved for the therapy of hypertension, guanfacine is currently indicated for the treatment of attention deficit hyperactivity disorder (ADHD) [37,38,39]. Guanfacine is a highly selective agonist of α_2A_ ADRs with very low affinity for other adrenergic receptors [40]. Guanfacine was proven to be more selective for the α_2A_ ADR subtype than clonidine, which also targets with high affinity to α_2B_, α_2C_, and imidazoline receptors [41,42]. Guanfacine preferentially binds to postsynaptic α_2A_ receptors, which mainly mediate its beneficial cognitive effects in the prefrontal cortex (PFC) [41,43]. Guanfacine demonstrated to improve the PFC function by strengthening PFC network connections through the inhibition of the cAMP-potassium channel signaling in postsynaptic spines [44,45]. Compared to clonidine, guanfacine has a weaker presynaptic action at α_2A_ receptors, and therefore it shows a better tolerability, producing hypotensive and sedative effects, but at a lower extent [46]. In fact, in animal studies, low doses (0.5–1 mg/Kg) of guanfacine improved working memory without reducing blood pressure or causing significant sedation, whilst only higher doses (10 mg/Kg) provoked more relevant adverse effects [47].

In light of these considerations, we deemed it interesting to investigate the drug guanfacine as an attractive TAAR1-targeting lead compound and provide it as a template molecule for further chemical improvements. We explored the in vitro functional activity of guanfacine at *h*TAAR1 receptors, while in vivo follow-up studies showed that guanfacine decreased the locomotor activity of DAT-KO rats.

## 2. Results and Discussion

### 2.1. Probing In Silico S18616 as Dual TAAR1 and α_2_-ADR Ligands

To pursue useful information for the design and discovery of novel selective *h*TAAR1 or α_2_-ADR ligands, it was deemed interesting to explore in silico the previously mentioned dual agonist **S18616**. Initially, the structural information of the two biological targets was compared by superimposition of their 3D structures. The *h*TAAR1 model (AF-Q96RJ0-F1) by the AlphaFold protein structure database [48,49] and the α_2_-ADR X-ray structure (PDB code = 6KUY) [50] were taken into account (see method section for details) and aligned via Blosum62 (MOE software- 2019.01 version). The reliability of the two GPCRs alignment can be assessed by the values of the pairwise percentage residue identity (PPRI; PPRI > 22%) and similarity (PPRS; PPRS > 38%), as shown in Figure S1.

As regards the 6KUY co-crystallized agonist, the experimental positioning suggests key contacts with Asp113 and π-π or cation-π stacking with the surrounding aromatic residues Phe390, Phe391, Trp387, and Phe412 (see Figure S2). This piece of information allows the corresponding protein cavity to be derived at the superposed TAAR1 receptor.

As shown in Table 1, the putative TAAR1 binding site includes Asp103, Ser198, Trp264, Phe267, Phe268, and Tyr294 as conserved residues. On the contrary, the *h*TAAR1 hydrophobic residues Ile104, Val184, and Ile290 are in place of the α_2_-ADR Val114, Ile190, and Phe412, while the *h*TAAR1 Ser107 corresponds to the α_2_-ADR Cys117.

Accordingly, the choice of the mostly hydrophobic structures is expected to turn in dual acting derivatives while the introduction of polar moieties would allow more selective *h*TAAR1 ligands to be designed.

On this basis, molecular docking studies involving the prototype **S18616** were performed to better clarify: (i) its different positioning as a dual agonist at *h*TAAR1 and α_2_-ADR and to (ii) be exploited as a control compound for the following molecular docking simulations of the imidazoline/imidazole (**5a-5e, 6a-6e, 11-54**), herein investigated. Details of the calculated scoring functions as shown for both **S18616** and the compounds **5a-5e, 6a-6e, 11-54** are listed in Tables S1 and S2.

**Figure 3:**
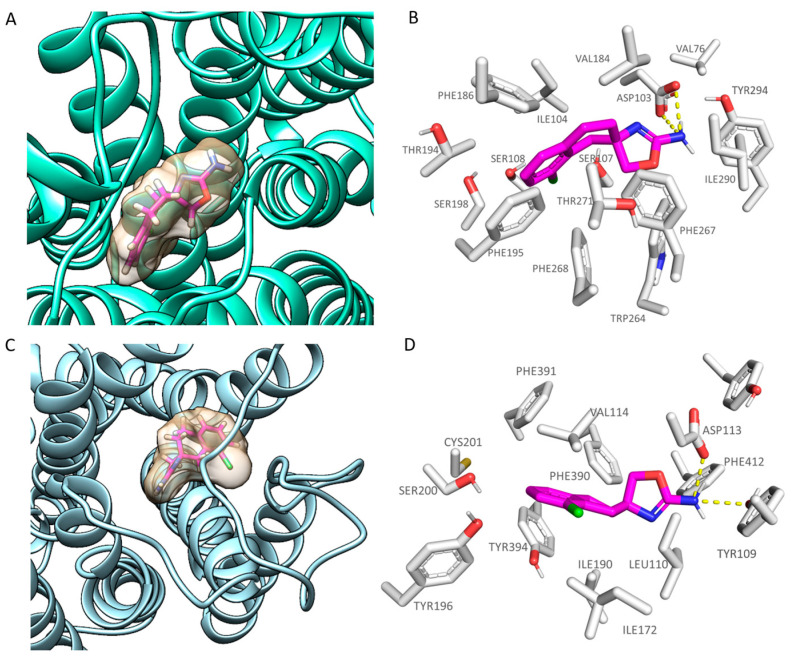
Docking mode of **S18616** (C atom; magenta) at the *h*TAAR1 (**A**,**B**) and α_2_-ADR (**C**,**D**) binding site. A perspective of the ligand volume (light brown) and of the protein cavity is depicted in (**A**) and (**C**), respectively. The most important residues involved in the agonist binding are labelled (**B**,**D**). **A** and **C** representations have been performed via Chimera 1.16 [51], while the ligand-protein contacts have been explored by means of PyMol software 2.5.2—Incentive Product Copyright (C) Schrodinger, LLC [52].

As shown in Figure 3A,C, **S18616** featured small dimensions, which allowed the compound to occupy both the *h*TAAR1 and α_2_-ADR ligand binding site, respectively. As shown in Figure 3B, **S18616** was engaged in H-bonds with Asp103 within the *h*TAAR1 binding pocket, while the spirocyclic system was projected towards Ile104, Val184, Phe186, Phe195, and Phe267, featuring Van der Waals contacts and π-π stacking.

The docking positioning of the dual acting compound **S18616** at the α_2_-ADR cavity pointed out additional H-bonds, involving the amino group of the agonist and the key residues Asp113 and Tyr109 (see Figure 3D). Notably, this information agreed with the higher potency trend of this compound towards α_2_-ADR (EC_50_ = 0.7 nM) with respect to *h*TAAR1 (EC_50_ = 15 nM). Furthermore, the spirocyclic system of the ligand experienced π-π stacking and Van der Waals contacts with the surrounding residues Val114, Phe390, Phe391, and Tyr394. A complete view of **S18616** at the whole *h*TAAR1 and α_2_-ADR (PDB code 6KUY) proteins is shown in Figure S4.

### 2.2. Comparative Molecular Docking of Dual Acting Compounds

With the aim of exploring the main features turning in *h*TAAR1 and α_2_-ADR targeting ability, we explored via molecular docking studies the previously mentioned imidazoline- and imidazole-containing derivatives (**5a-5e, 6a-6e, 11-54**) [35] at both the two proteins (see details of the calculated scoring functions in Tables S1 and S2). Thus, 45 compounds were considered, including the imidazoline compounds (**5a**-**5e**) and the imidazole analogues (**6a**-**6e**, **11**-**54**) (see Table S3 for the chemical structure and biological activity).

The imidazoline series, group **5a**-**5d** (*h*TAAR1 Ki = 82–1640 nM α_2_-ADR Ki = 25–204 nM) proved to be endowed with higher affinity and selectivity towards α_2_-ADR over *h*TAAR1. Conversely, the corresponding congeners **6a**-**6d** showed higher affinity and selectivity for *h*TAAR1. The unsubstituted derivative **5a** was the less potent of this series, featuring *h*TAAR1 Ki values = 1640 nM, gaining promising affinity towards the α_2_-ADR (Ki = 98.4 nM).

**Figure 4:**
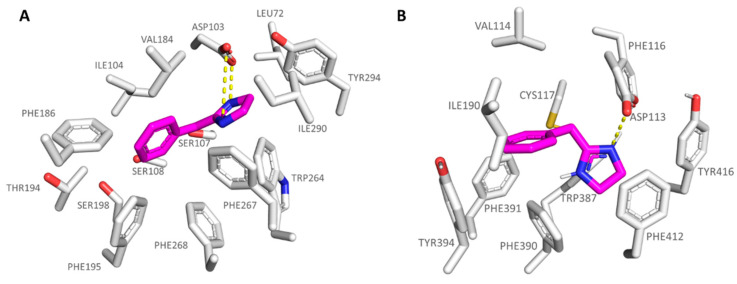
Docking pose of compound **5a** (C atom; magenta) within the *h*TAAR1 (**A**) and α_2_-ADR (**B**) binding sites. The most important residues involved in the agonist binding are labelled. Ligand-protein contacts have been explored by means of PyMol software 2.5.2—Incentive Product Copyright (C) Schrodinger, LLC [52].

Docking results at the TAAR1 model reported variable docking poses, probably due to the absence of any substituent at the **5a** phenyl ring. As a result, the compound was weakly stabilized at the receptor binding site, featuring the required H-bond with Asp103 by one of the nitrogen atoms of the imidazoline ring (see Figure 4A).

The heterocycle core was also projected towards the Trp264, Phe267, and Tyr294, detecting π-π stacking and cation-π contacts. The benzyl moiety was surrounded by the hydrophobic residues Ile104, Phe186, Phe154, and Phe195. Conversely, the **5a** docking positioning at the α_2_-ADR revealed a maintained H-bond with the conserved Asp113 via the imidazoline ring, while the benzyl core was engaged in π-π stacking and Van der Waals contacts with Phe390, Tyr395, and Val114, Ile 190, respectively (see Figure 4B).

**Figure 5:**
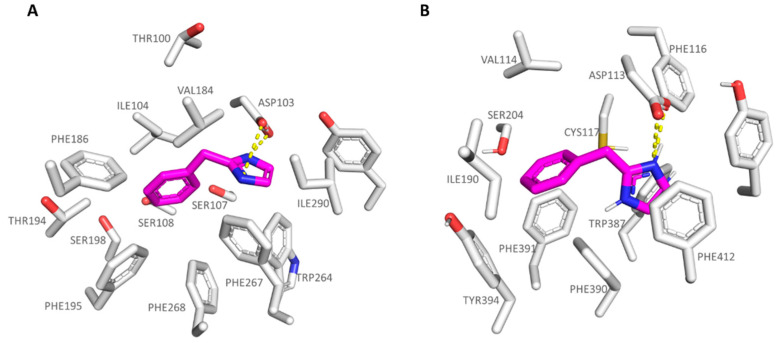
Docking pose of compound **6a** (C atom; magenta) within the *h*TAAR1 (**A**) and α_2_-ADR (**B**) binding sites. The most important residues involved in the agonist binding are labelled. Ligand-protein contacts have been explored by means of PyMol software 2.5.2—Incentive Product Copyright (C) Schrodinger, LLC [52].

The imidazole-containing analogue **6a** (*h*TAAR1 Ki = 400 nM, α_2_-ADR Ki = 1880 nM), as a selective TAAR1-targeting compound, was H-bonded to the *h*TAAR1 Asp103, moving the imidazole ring in proximity of Ser207, Trp264, and Phe267 (see Figure 5A). This kind of positioning was also allowed by the net of Van der Waals contacts stabilizing the benzyl pendant near Ile104.

Interestingly, **6a** displayed a comparable docking mode also at the α_2_-ADR, being on the other hand weakly stabilized at the receptor cavity due to a low number of π-π contacts with the surrounding residues, and lack of polar contacts with the previous Ser207, herein Cys117; thus, **6a** experiences lower affinity values for α_2_-ADR. Conceivably, it can also be explained by the presence of a valine residue (Val114) instead of an isoleucine in *h*TAAR1 (Ile104) in tandem with the deeper crevice of α_2_-ADR compared to *h*TAAR1 (Figure 5B).

**Figure 6:**
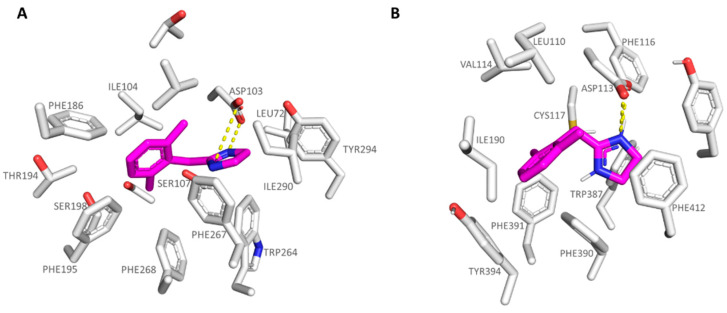
Docking pose of compound **5c** (C atom; magenta) within the *h*TAAR1 (**A**) and α_2_-ADR (**B**) binding sites. The most important residues involved in the agonist binding are labelled. Ligand-protein contacts have been explored by means of PyMol software 2.5.2—Incentive Product Copyright (C) Schrodinger, LLC [52].

The introduction of bulkier substituents onto the benzyl group, such as those featured by **5b**-**5d** (α_2_-ADR Ki = 25–204 nM; *h*TAAR1 Ki = 82–500 nM) and **6b**-**6d** (*h*TAAR1 Ki = 24–1390 nM; α_2_-ADR Ki = 162–2400 nM), ameliorated the compound affinity and selectivity towards the α_2_-ADR and TAAR1 proteins, respectively. In particular, the effective compound **5c** was H-bonded to the *h*TAAR1 and α_2_-ADR key residue Asp103 and Asp113, respectively, while the dimethyl-benzyl moiety was stabilized at the receptors cavity via Van der Waals contacts with: (i) Ile104, Val184, Phe186, and Phe195 at TAAR1 and (ii) Ile110, Val114, Phe390, and Phe391 at α_2_-ADR (see Figure 6).

In addition, the **5c** five-membered ring projected towards Tyr294 and Phe267 in *h*TAAR1 and Trp387, Phe390, and Tyr416 in α_2_-ADR, detecting cation-π contacts with the surrounding aromatic residues.

**Figure 7:**
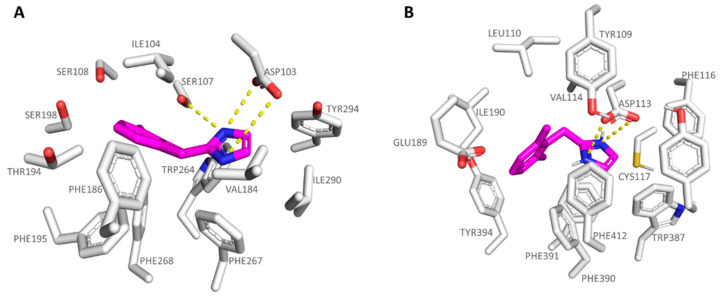
Docking mode of **6c** (C atom; magenta) at the *h*TAAR1 (**A**) and at the α_2_-ADR (**B**) binding site. The most important residues involved in the agonist binding are labelled. Ligand-protein contacts have been explored by means of PyMol software 2.5.2—Incentive Product Copyright (C) Schrodinger, LLC [52].

As shown in Figure 7A, this kind of positioning was also better guaranteed by the analogue **6c** as the *h*TAAR1 agonist (**6c** *h*TAAR1 Ki = 36 nM), featuring higher *h*TAAR1 affinity values if compared to **5c**, than as the α_2_-ADR targeting compound (**6c** α_2_-ADR Ki = 162 nM). At the α_2_-ADR, compound **6c** experienced a limited number of π-π and cation-π stacking with the aforementioned Trp387, Phe390, and Tyr416, moving the terminal benzyl group much more in proximity of Ile110, Val114, and Ile190 (see Figure 7B).

Synthetic efforts described in the literature [35] were used to identify more potent TAAR1 targeting ligands, maintaining the aromatic imidazole ring instead of the previous imidazoline core as the main five-membered ring. Different substitutions at the previous benzyl group were also afforded in order to explore in more detail the SAR activity of this series of compounds.

Structural elongation of 4-substituted imidazole-based compounds such as the **13**-**39** derivatives (*h*TAAR1 Ki = 2–195 nM; α_2_-ADR Ki = 14.30–3040 nM) led to more potent and selective *h*TAAR1 agonists over α_2_-ADR. Conversely, most of the investigated 2-substituted imidazoles (**49-54**; *h*TAAR1 Ki = 68–4250 nM; α_2_-ADR Ki = 254–3760 nM) were endowed with higher selectivity towards the α_2_-ADR protein.

**Figure 8:**
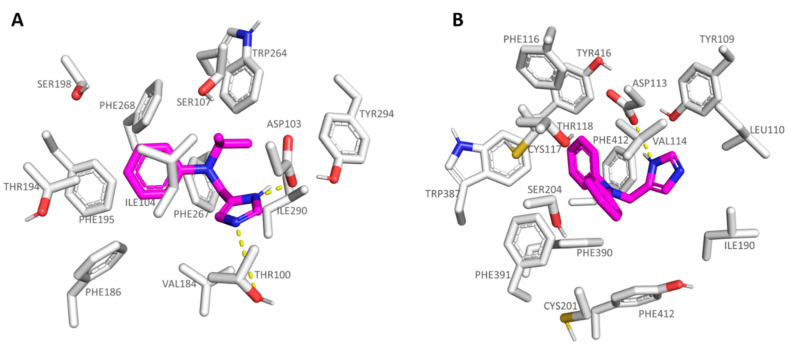
Docking positioning of **16** (C atom; magenta) at the *h*TAAR1 binding site (**A**) and of **18** at the α_2_-ADR cavity (**B**). The most important residues involved in the agonist binding are labelled. Ligand-protein contacts have been explored by means of PyMol software 2.5.2—Incentive Product Copyright (C) Schrodinger, LLC [52].

As regards the agonists **13**-**19** series, bearing the *N,N*-disubstituted aniline group, all of them were potent TAAR1 agonists, with **16** (*h*TAAR1 Ki = 22 nM; Ki α_2_-ADR Ki = 521 nM) being the most promising and selective derivative within the whole series. On the contrary, compound **18** (*h*TAAR1 Ki = 69 nM; Ki α_2_-ADR Ki = 62 nM) experienced comparable affinity towards the two biological targets. Based on our docking studies, **16** was engaged in H-bonds with Thr100 and Asp103 thanks to the imidazole nitrogen atoms, while the branched and hydrophobic isopropyl group was surrounded by the narrow pocket including Ile104 and Trp264. As a consequence, the aniline aromatic ring efficiently displayed π-π stacking with Phe186, Phe195, and Phe268 (see Figure 8A).

Conversely, compound **18,** bearing a flat rigid phenyl group onto the aniline moiety, was H-bonded to Asp113 in α_2_-ADR, moving the previously mentioned aromatic ring towards the deep receptor cavity delimited by Val114, Val197, and Phe391 (see Figure 8B). On this basis, it is thought that the introduction of small, folded groups (such as the **16** isopropyl group) as pendants at the aniline nitrogen atom would be better arranged within the *h*TAAR1 cavity than at α_2_-ADR. On the contrary, bulkier and rigid groups such as the phenyl ring of **18** would be better stabilized within the deeper α_2_-ADR binding site.

Accordingly, the branched **16** and **17** analogues (*h*TAAR1 Ki = 22–32 nM; Ki α_2_-ADR Ki = 128–521 nM) displayed higher selectivity for *h*TAAR1 than the agonists **18** and **19** (*h*TAAR1 Ki = 69–110 nM; Ki α_2_-ADR Ki = 62–330 nM).

**Figure 9:**
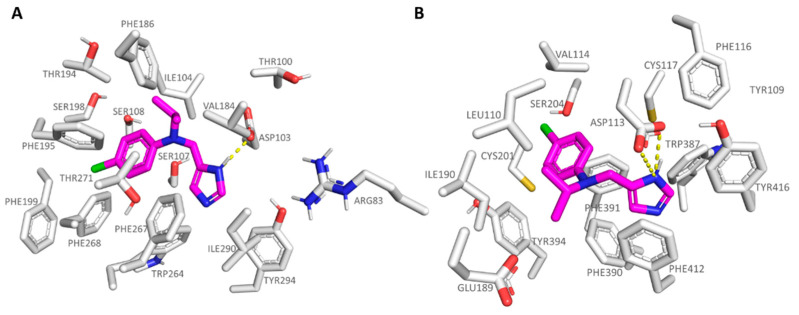
Docking mode of **24** (C atom; magenta) at the *h*TAAR1 (**A**) and at the α_2_-ADR (**B**) binding site. The most important residues involved in the agonist binding are labelled. Ligand-protein contacts have been explored by means of PyMol software 2.5.2—Incentive Product Copyright (C) Schrodinger, LLC [52].

In accordance with this information, ring cyclization on the previous aniline nitrogen atom led to effective TAAR1 and α_2_-ADR ligands, such as **20**-**21** (*h*TAAR1 Ki = 12–35 nM; α_2_-ADR Ki = 4.90–48 nM) endowed with a proper hydrophobic core as the bicyclic ring. To pursue the design of selective *h*TAAR1 targeting compounds, a new series of imidazoles was reported by focusing on the previously described effective isopropyl group in presence of further moieties at the aniline aromatic portion. The **22**-**35** series (*h*TAAR1 Ki = 2–128 nM; α_2_-ADR Ki = 14.30–3040 nM) included a halogenated aniline ring in the presence of the isopropyl group or *tert-*butyl substituent, leading in any case to effective and selective *h*TAAR1 ligands. As shown in Figure 9A, compound **24** (*h*TAAR1 Ki = 6 nM; α_2_-ADR Ki = 840 nM) properly moved the imidazole core near Asp103, while the branched aliphatic substituent was surrounded by Ile104 and Phe186. On the other hand, the terminal halogenated phenyl ring was engaged in π-π stacking and polar contacts with the aromatic Phe199, Trp264, and Phe268.

In the case of the α_2_-ADR protein, compound **24** maintained the mandatory key contact with Asp113, while the *p*-Cl-phenyl ring proved to be lacking the π-π stacking with aromatic residues, being oriented towards Ser204 and Val114 (Figure 9B). Conversely, the *m*-Cl substituted analogue **23** (α_2_-ADR Ki = 288 nM) efficiently placed the aromatic ring near Trp387 and Phe412, gaining π-π contacts.

The introduction of the heterocyclic ring instead of the aniline moiety, as shown by the **36**-**39** series (*h*TAAR1 Ki = 12–195 nM; α_2_-ADR Ki = 252–3300 nM), proved to be beneficial, leading to the discovery of very selective TAAR1 ligands endowed with high affinity values for the desired receptor. Indeed, compound **37** (*h*TAAR1 Ki = 12 nM; α_2_-ADR Ki = 74 nM) displayed additional H-bonds with Thr100 and Ser107, thanks to the imidazole ring and the nitrogen atom of the pyridine substituent Figure S5.

Beyond the reported SAR data, most of the investigated 2-substituted imidazoles (**49-54**; *h*TAAR1 Ki = 68–4252 nM; α_2_-ADR Ki = 254–4352 nM) were more selective for the α_2_-ADR protein than *h*TAAR1, except for compound **53** (*h*TAAR1 Ki = 68 nM; α_2_-ADR Ki = 4352 nM). As shown in Figure S6A, this compound maintained the required key contact with Asp103, while the imidazole ring and the terminal phenyl core detected π-π stacking with Trp264, Tyr294, Phe186, and Phe195, respectively.

On the other hand, the analogues **50** and **51** (*h*TAAR1 Ki = 710–1270 nM; α_2_-ADR Ki = 254–497 nM) were better stabilized at the α_2_-ADR protein via Van der Waals contacts, involving the aliphatic substituent onto the aniline nitrogen atom and Val114 and Cys117 being the imidazole ring H-bonded to Asp113 (Figure S6B).

### 2.3. QSAR Analyses and Pharmacophore Modeling

In the search for new bioactive compounds, the computational methods, including quantitative structure–activity relationship (QSAR) analyses, represent a useful tool to predict the potency, the selectivity, and also the cytotoxicity profile of known and novel compounds [30,53].

In this context, we proceeded with the development of two QSAR models to identify the compound structural features influencing *h*TAAR1 (model A) or α_2_-ADR (model B) binding affinity as experienced by dual acting compounds. These derivatives were collected by the literature [35,36] and referred to the previously mentioned imidazole- and imidazoline-containing derivatives shown in Table S3.

The two QSAR models were built considering the compound positioning observed by docking calculations. The results are expected to be a useful tool for the preliminary evaluation of further novel analogues and to guide the development of a new series of derivatives.

In detail, about 300 molecular descriptors (2D and 3D parameters) were calculated by means of MOE2019.01 software [54].

The bidimensional (2D) descriptors include seven groups, regarding physical properties (2D-I), subdivided surface areas (2D-II), atom and bond counts (2D-III), connectivity-based descriptors (2D-IV), partial charges descriptors (2D-V), pharmacophore features descriptors (2D-VI), and the so-called adjacency and distance matrix descriptors (2D-VII). Concerning 3D-descriptors, these are divided into five groups, including the potential energy (3D-I), MOPAC (3D-II), surface area (3D-III), volume and shape (3D-IV), and conformation-dependent charge descriptors (3D-V). In this study, the choice of the most relevant descriptors to explain the bioactive behavior (within model A and B) was pursued, applying a statistical approach previously described [55].

For each model, the corresponding final equation was derived by splitting the compounds into a training and a test set using the Kennard–Stone design [56], one of the most exploited algorithms for guiding the choice of a subset of samples with a distribution as close as possible to the uniform distribution. In particular, the Kennard–Stone algorithm was applied, adding the response vector (*h*TAAR1 pKi in model A and α_2_-ADR pKi in model B) as a further column to the matrix of the collected descriptors in order to guarantee that the training set compounds were distributed evenly within not only in the space described by the descriptors, but also by the response values [55].

Among the 333 molecular descriptors, the most informative ones were identified using a multivariate variable selection method. In particular, iterative stepwise elimination PLS (ISEPLS) [57] was applied to evaluate the relevance of the predictors with regard to the possibility of predicting the response variable y (pKi; *h*TAAR1 in model A and α_2_-ADR in model B). Following this approach, 10 and 11 descriptors were retained for model A and model B, respectively, as described in the following section.

#### 2.3.1. QSAR Model A—*h*TAAR1 Binding Affinity

Based on the procedure described above, model A was derived taking into account 10 descriptors selected by using ISEPLS as a multivariate variable selection method, including most of them with 3D parameters. Indeed, six of the selected molecular descriptors fall in the 3D cluster, while the other ones in the 2D. Details of the selected descriptors as well as their relevance in the developed QSAR model discussed as follows, are shown in Table 2.

The regression model was calculated using PLS analysis and dividing the whole dataset of 45 compounds into a training set of 31 molecules (**5b**-**5e**, **6a**, **6c**, **6d**, **12**, **13**, **15**, **16**, **18**-**20**, **22**, **24**, **26**-**29**, **31**-**35**, **37**, **39**, **50**-**53**) used to develop the QSAR model and into a test set, including 14 derivatives (**5a**, **6b**, **6e**, **11**, **14**, **17**, **21**, **23**, **25**, **30**, **36**, **38**, **49**, **54**) to evaluate the reliability of the regression model. The final PLS model gave a cross validated r^2^ (r^2^cv) = 0.74, a non-cross validated r^2^ (r^2^n_cv_) = 0.85, root mean square error (RMSE) = 0.282, and a test set r^2^ (r^2^_pred_) = 0.65. The predicted and experimental *h*TAAR1 Ki for all the compounds is reported as tables, along with the collected descriptors, as shown in Table S4.

**Figure 10:**
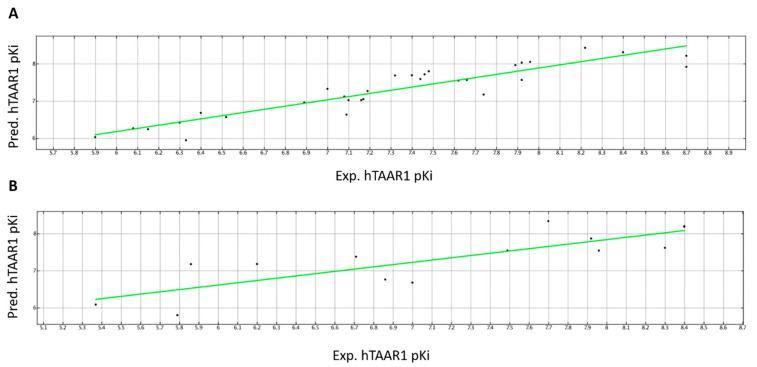
Distribution of the predicted (Pred. *h*TAAR1 pKi) versus the experimental (Exp. *h*TAAR1 pKi) *h*TAAR1 binding affinity featured by the training set (**A**) and test set derivatives (**B**). Compounds are represented as dots.

A perspective of the performance featured by this model is reported in Figure 10, as distribution of the predicted *h*TAAR1 affinity values (Pred.pKi), with respect to the experimental ones (Exp.pKi), of the training set and test set compounds.

Quantitatively, the role played by the selected descriptors with respect to the TAAR1 targeting ability is explained by the following equation (Equation (1)).

Predicted *h*TAAR1 Ki = 5.88651 −0.78177 * GCUT_SMR_0 −0.02455 * E +2.02712 * DipoleY +0.80749 * DipoleZ −0.00279 * DCASA −0.96171 * Q_RPC −1.76365 * Q_VSA_FHYD +0.13838 * SlogP_VSA4 −0.69487 * vsurf_EDmin1 +0.30105 * vsurf_IW5. (1)

#### 2.3.2. QSAR Model B—α_2_-ADR Binding Affinity

Regarding the α_2_-ADR regression model, the model B was developed using 11 descriptors, selected by ISEPLS; these descriptors include most of the 2D parameters. Indeed, seven of the selected molecular descriptors were in the 2D cluster, while the other ones in the 3D. Details of the selected descriptors in tandem with their importance in the developed QSAR model are discussed as follows and shown in Table 3.

The regression model B was developed using PLS analysis on the 31 compounds assigned to the training set (**5b**-**5e**, **6a**, **6c**, **6d**, **12**, **13**, **15**, **16**, **18**-**20**, **22**, **24**, **26**-**29**, **31**-**35**, **37**, **39**, **50**-**53**) in order to develop the QSAR model, while the other 14 included in the test set (**5a**, **6b**, **6e**, **11**, **14**, **17**, **21**, **23**, **25**, **30**, **36**, **38**, **49**, **54**) were used to assess the reliability of the regression model. The final PLS model provided a cross validated r^2^ (r^2^cv) = 0.43, a non-cross validated r^2^ (r^2^n_cv_) = 0.80, root mean square error (RMSE) = 0.358, and a test set r^2^ (r^2^_pred_) = 0.55. The predicted and experimental α_2_-ADR Ki for all the compounds is reported as tables, along with the collected descriptors, as shown in Table S5.

**Figure 11:**
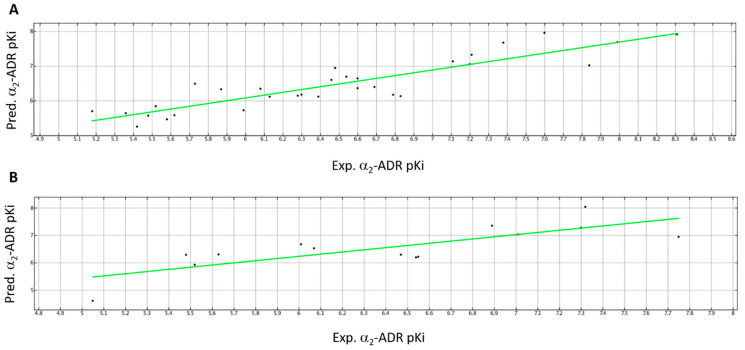
Distribution of the predicted (Pred. α_2_-ADR pKi) *versus* the experimental (Exp. α_2_-ADR pKi) α_2_-ADR binding affinity featured by the training set (**A**) and test set derivatives (**B**). Compounds are represented as dots.

A perspective of the performance featured by this model is reported in Figure 11, as distribution of the predicted α_2_-ADR affinity values (Pred.pKi), with respect to the experimental ones (Exp.pKi), of the training set and test set compounds.

Quantitatively, the role played by the selected descriptors to affect the α_2_-ADR targeting ability is explained by the following equation (Equation (2)).

Predicted α_2_-ADR pKi = 7.00986 +0.56459 * GCUT_SMR_1 −1.49103 * balabanJ −0.02896 * E_tor +0.04376 * Q_VSA_FHYD −0.08643 * Q_VSA_PNEG +0.06695 * Q_VSA_POL +0.02122 * vsa_other +0.03314 * SlogP_VSA3 +0.32188 * vsurf_ID1 +1.04420 * vsurf_ID7 +0.29374 * vsurf_IW4 (2)

By a comparison of the two models, *h*TAAR1 binding affinity values increase in the presence of polar ligands (via DipoleY parameter) and of a limited number of positively charged groups in order to build a chemical structure characterized by a similar charge delocalization onto the whole molecule (see DCASA descriptor). This could be achieved by the choice of *p*-substituted phenyl rings instead of *o*-substituted analogues. Accordingly, compounds **24** (*h*TAAR1 Ki = 8.22 M, DipoleY = 0.2702; DCASA =23.7212) and **29** (*h*TAAR1 Ki = 7.89 M, DipoleY= 0.2724; DCASA= 155.0814) show higher Ki values than the corresponding congener **22** (*h*TAAR1 Ki = 7.40 M, DipoleY = 0.1838; DCASA = 57.1006) and **27** (*h*TAAR1 Ki = 7.74 M, DipoleY = 0.0533; DCASA = 160.3320).

This kind of substitution should be accompanied by hydrophobic properties, as featured by branched substituents rather than by flexible groups, which should be discouraged (as suggested by the Q_VSA_FHYD descriptor).

Conversely, the α_2_-ADR targeting ability could be improved by the introduction of polar and positively charged moieties or electron-donor features, as suggested by the Q_VSA_PNEG descriptor and Q_VSA_POL. Thus, the introduction of electron-rich atoms is discouraged. Accordingly, the α_2_-ADR agonist **12** (a_2_-ADR Ki = 7.49 M; Q_VSA_PNEG = 0.1369) featuring an alkyl-based spacer between the two terminal rings is endowed with higher Ki values than the corresponding amino-containing **13** (α_2_-ADR Ki = 6.46 M; Q_VSA_PNEG = 0.2738). The presence of an overall hydrophobic structure is in any case encouraged (Q_VSA_FHYD); the choice of flexible or extended substituents is preferred to branched groups, as suggested by the balabanJ and E_tor descriptors. On this basis, the choice of the ethyl-based spacer as experienced by **12** (α_2_-ADR Ki = 7.49 M; balabanJ = 1.6145; Q_VSA_FHYD = 0.9532; E_tor = 0.0625) rather than the methyl one as featured by **11** (α_2_-ADR Ki = 7.30 M; balabanJ = 1.7164; Q_VSA_FHYD = 0.9484; E_tor = 3.3058), seems to be encouraged, allowing for an extended ligand positioning. Notably, all this information proved to agree with those previously observed through docking studies, supporting the development of imidazoline and imidazole derivatives as more suited to α_2_-ADR and *h*TAAR1 ligands with respect to other heterocycles.

**Figure 12:**
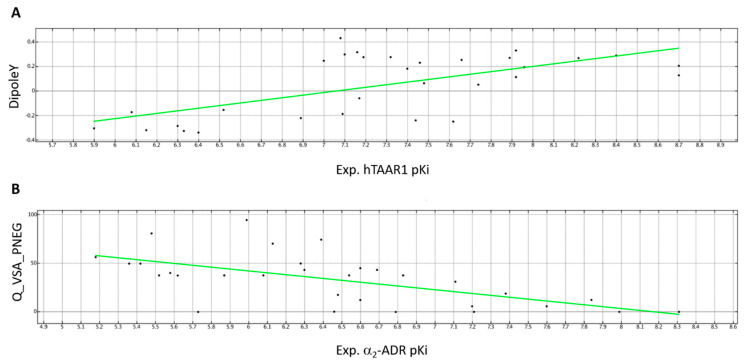
Schematic representation of the importance played by the DipoleY and Q_VSA_PNEG descriptors to influence the compound (*h*TAAR1 and α_2_-ADR) binding affinity values. The two descriptors are endowed by the highest RI values in model (**A**,**B**), respectively. Compounds are represented as dots.

A schematic representation of the role played by the most relevant descriptors affecting *h*TAAR1 (DipoleY, RI = 1.0000) and α_2_-ADR (Q_VSA_PNEG, RI = 1.0000) binding affinity, respectively, is reported in Figure 12.

A comparison of the opposite role played by the only common descriptor herein described in both the two models A and B (Q_VSA_FHYD) is shown in Figure S7.

#### 2.3.3. Pharmacophore Modeling

As a perspective of the molecular docking results and the QSAR analyses, the rational design of hydrophobic structures is expected to turn in dual acting *h*TAAR1 and α_2_-ADR derivatives while the introduction of polar moieties with a moderate number of positive-charged centers would allow more selective *h*TAAR1 ligands to be designed. Conversely, enrichment of positive-charged moieties onto the hydrophobic main core should improve the α_2_-ADR agonism ability. Notably, these data are in line with our previous findings concerning the TAAR1 pharmacophore model (PM), as determined on a set of oxazoline derivatives [31]. Indeed, the presence of a bulky aromatic ring tethered to a further hydrophobic core, bearing H-bonding features, was reported by us as pivotal to achieve the TAAR1-targeting ability.

On this basis, herein we reported novel PMs developed on these dual acting *h*TAAR1 and α_2_-ADR agonists, namely PM_A (*h*TAAR1) and PM_B (α_2_-ADR). PM_A included the most promising *h*TAAR1 agonists (pKi > 8 M) such as **12**, **23**-**26**, **30**, and **34**, while PM_B was derived based on the most potent α_2_-ADR ligands (pKi > 7.5 M), namely **5b**, **12**, **14**, **20**, and **33**.

**Figure 13:**
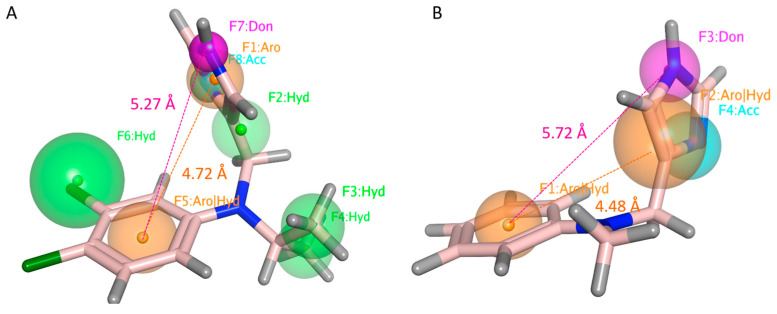
PM_A (*h*TAAR1 targeting ability) (**A**) and PM_B (α_2_-ADR targeting ability) (**B**) as featured by the dataset compounds. The most relevant features (F) are represented as colored spheres and classified as related to aromatic or hydrophobic groups (AroǀHyd) or only hydrophobic- (Hyd) or aromatic- cores (Aro), or to H-bonding acceptor (Acc) or donor (Don) groups. Compounds **25** (C atom; light pink) and **14** (C atom; light pink) were taken as representative of the dataset in PM_A and PM_B, respectively. Distance among the recurrent features shared by both PM_A and PM_B are reported.

For each model, only the pharmacophore features (F) shared by all the selected compounds was reported as aromatic or hydrophobic groups (AroǀHyd) or only hydrophobic- (Hyd) or aromatic- cores (Aro), or H-bonding acceptor (Acc) or donor (Don) groups. As shown in Figure 13, compounds **25** (*h*TAAR1 pKi = 8.4 M) and **14** (*h*TAAR1 pKi = 7.75 M) were taken as reference in PM_A and PM_B because of their promising affinity values, respectively.

As regards PM_A, the presence of two aromatic or hydrophobic cores (F1:Aro and F5:AroǀHyd) is required in the imidazole/imidazoline-based TAAR1 ligand, such as **25**, within a distance of 4.27 Å (Figure S8A). In detail, F1:Aro should be enriched with H-bonding features as exemplified by F7:Don and F8:Acc, placed at 2.22 Å to each other. In particular, the donor moiety is required for TAAR H-bonding via Asp103 residue. In PM_A, F7:Don should be located 5.27 Å from the terminal F5:AroǀHyd group. Interestingly, the introduction of additional hydrophobic substituents is required to gain promising *h*TAAR Ki values, as shown by the F2:Hyd, F3:Hyd, and F4:Hyd features. While F2:Hyd represents a flexible spacer to allow the required distances between the previously cited F1:Aro and F5:AroǀHyd, F3:Hyd and F4:Hyd guarantee a proper steric hindrance, which is thought to be beneficial to: (i) interact with the *h*TAAR1 hydrophobic cavity and (ii) to oblige the agonist positioning towards a “Y-shape” folded conformation, as it is in the case of other GPCRs ligands [61]. In the case of the *h*TAAR1 PM_A, F3:Hyd and F4:Hyd are placed 4.83 Å and 5.58 Å from F1:Aro and 4.70 Å, 4.92 Å from F5:AroǀHyd.

Among the aforementioned features, the choice of two terminal aromatic/hydrophobic features in tandem with H-bonding moieties is maintained to allow the a_2_-ADR binding ability (Figure S8B). Accordingly, the α_2_-ADR ligand exhibits two aromatic or hydrophobic features (F1:AroǀHyd and F2:AroǀHyd) placed at 4.48 Å to each other, as shown by the agonist **14** imidazole and phenyl rings, being the F3:Don and F4:Acc nearby (2.19 Å). The required F3:Don feature achieving polar contacts with Asp113 is located 5.72 Å far from F1:AroǀHyd.

Bearing in mind this information, the α_2_-ADR agonist guanfacine (EC_50_ ~ 25.1 nM [62]) underwent biological evaluation as a putative dual acting agent, also targeting *h*TAAR1. The drug guanabenz, already known as an α_2_-ADR (EC_50_ ~ 16.32 nM [63]) and *m*TAAR1 agonist (EC_50_ = 90 nM [64]), was also tested at *h*TAAR1 for comparative purposes. Indeed, both the two compounds fulfilled the previously mentioned minimum qualitative criteria to achieve *h*TAAR1 and/or α_2_-ADR binding ability (see the chemical structure in Figure 13). While the dicloro-substituted phenyl ring of the two compounds guarantees the proper ligand folding, the terminal positively charged moiety should support the dual acting ability of the compounds.

Details of the following in vitro/in vivo assays are reported as follows.

### 2.4. Guanfacine and Guanabenz Are TAAR1 Agonists

**Figure 14:**
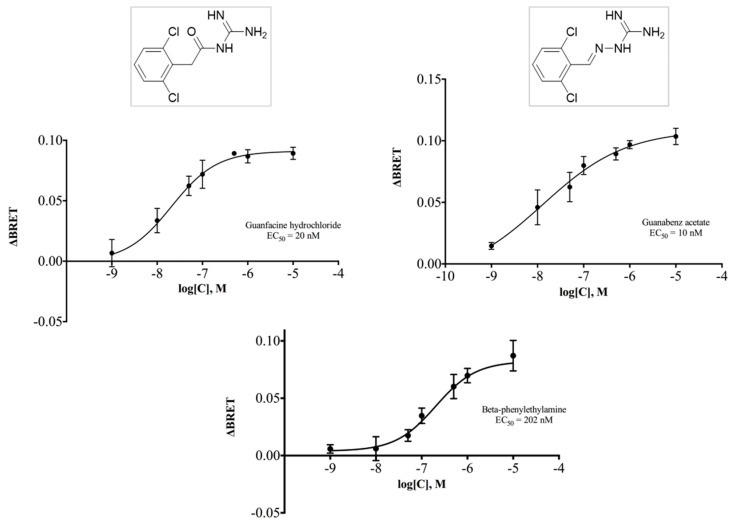
The functional activity at *h*TAAR1 of guanfacine, guanabenz, and β-PEA presented as concentration-dependent curves.

*h*TAAR1 is coupled to stimulatory G proteins and thus its activation induces an increase in the cAMP production. We measured the potential activity of guanfacine and guanabenz by using a BRET-based assay [65] in which HEK293 cells were transfected with *h*TAAR1, or the empty vector as control, and the cAMP BRET biosensor. The standard TAAR1 agonist β-PEA was used as a reference compound, as in our tests it also increased cAMP through TAAR1 activation (EC_50_ = 202 nM). A dose-response experiment was performed using concentrations ranging from 1 nM to 10 μM to calculate the corresponding EC_50_ and the Emax values. Both guanfacine and guanabenz displayed an Emax > 85% at *h*TAAR1, thus acting as full agonists (Figure 14) with similar EC_50_ in the low nanomolar range (guanfacine EC_50_ = 20 nM; guanabenz EC_50_ = 10 nM, see Figure 14).

Guanabenz was already described as a partial agonist at *m*TAAR1 (EC_50_ = 7 nM) and chimeric receptor *c*TAAR1 (EC_50_ = 25 nM), as a more responsive model of *h*TAAR1, in which the N-terminal, C-terminal, and third intracellular loop sequences of the human ortholog were replaced by the corresponding mouse sequences [66]. Successively, Lam et al. [64] observed the full agonist activity of guanabenz at *m*TAAR1 (EC_50_ = 90 nM), using a BRET cAMP reporter. Our data also validate the potent agonist activity of guanabenz at *h*TAAR1. The interest in guanabenz has been growing again due to its beneficial effects, not only in the circulatory system as a full agonist at the α_2A_-adrenoceptor, but also in other pharmacological settings. Recently, it showed a weight-reducing effect and the attenuation of some metabolic parameters in obese rats [63,67,68]. Activation of TAAR1 was found to provide beneficial effects on glucose control [69] and body weight in animal models of type 2 diabetes and obesity by incretin-like effects [70]. TAAR1/Gα_s_-mediated signaling pathways that promote insulin secretion, demonstrated an improvement in pancreatic β-cell function and proliferation [69]. Therefore, further investigations are warranted as a chance to bridge the gap between the beneficial influence of guanabenz on metabolic disturbances and its TAAR1-targeting ability.

It should be emphasized that both guanfacine and guanabenz caused the increase in the cAMP levels in cells co-transfected with *h*TAAR1 and the cAMP sensor, while activation of the α_2_-ADR-dependent signaling should have caused the opposite effect. This multidirectional action on cAMP levels should be considered when effects of drugs acting through both TA-AR1 and α_2_-ADR are evaluated.

### 2.5. Administration of Guanfacine Resulted in Decrease in Locomotor Activity of DAT-KO Rats

To evaluate the in vivo pharmacological effect of guanfacine, we used a rodent model of hyperdopaminergia, the dopamine transporter knockout (DAT-KO) rats, that also mimics some phenotypic aspect of ADHD and has certain predictive validity for the search of novel pharmacological agents to control hyperactivity and cognitive processes in patients with this disease [71,72]. In fact, guanfacine demonstrated significant positive effects in tests aimed at evaluating cognitive dysfunction of DAT-KO rats [73]. All TAAR1 agonists tested so far in DAT-KO animals also showed an inhibitory effect on spontaneous dopamine-dependent hyperactivity of these mutants [71]. Thus, the effect of guanfacine on hyperactivity of DAT-KO rats was evaluated.

**Figure 15:**
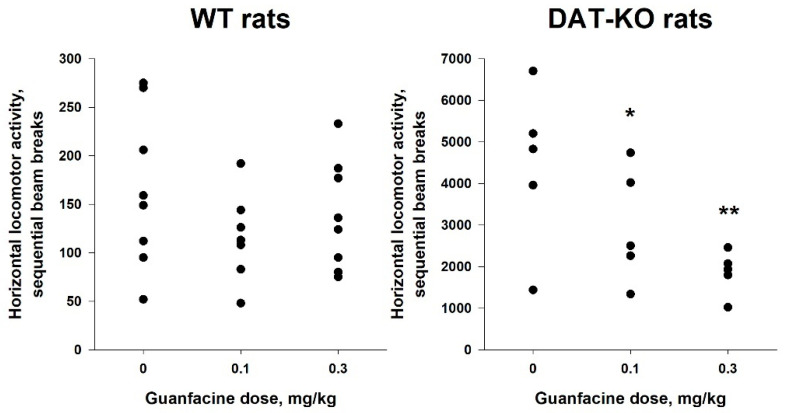
Action of guanfacine (0.1 and 0.3 mg/kg, i/p) on locomotor activity of WT and DAT-KO rats. ** *p* < 0.01, * *p* < 0.05 (Bonferroni’s test) vs. the vehicle-treated animals.

Results are presented on Figure 15. In strict concordance with previous works [71], DAT-KO rats demonstrated higher levels of locomotor activity than their WT littermates (4425.6 ± 868.67 vs. 164.8 ± 28.53; the U-test: *p* < 0.01). We used the mixed ANOVA (the within-subject factors ‘5-min bin’ (1–12) and ‘dose’ (0.0; 0.1; 0.3 mg/kg), the between-subject factor ‘mutation’ (‘WT’; ‘KO’); the random factor ‘rat #’) on rank-transformed data to analyze results. The pretreatment with guanfacine resulted in a decrease in locomotor activity (the effect of factor ‘dose’: F(2,220) = 11.32, *p* < 0.001). Locomotor activity of the rats decreased from the 1st to the 12th bin (the effect of factor ‘5-min bin’: F(11,51) = 2.51, *p* = 0.01). The lack of DAT was associated with an increased level of locomotor activity (the effect of factor ‘mutation’: F(1,11) = 222.94, *p* < 0.001). The mixed ANOVA revealed the statistically significant effect of interaction of the factors ‘dose’ and ‘mutation’ (F(2,220) = 8.45, *p* < 0.001) but not the factors ‘dose’, ‘mutation’, and ‘5-min bin’ (F(22,41) = 0.58, *p* = 0.92). We used Bonferroni’s test for *post hoc* comparisons. As presented in the figure, the pairwise comparisons revealed that guanfacine treatment was associated with decreased locomotor activity in DAT-KO (0.3 vs. 0.0 mg/kg: *p* < 0.001; 0.3 vs. 0.0 mg/kg: *p* = 0.02) but not in WT rats (0.3 vs. 0.0 mg/kg: *p* = 1.0; 0.1 vs. 0.0 mg/kg: *p* = 0.12). While the contribution of the α_2_-ADR-mediated action of guanfacine to this effect cannot be fully excluded, these data support a general concept of TAAR1 agonism counteracting excessive dopamine function [3,71].

## 3. Materials and Methods

### 3.1. Ligand and Protein Preparation

Each compound was built, parameterized (AM1 partial charges as the calculation method), and energy minimized within Molecular Operating Environment software (MOE) [54] (Energy Minimize tool) using the MMFF94x forcefield of MOE and RMS (root mean square) gradient equal to 0.0001, with the root mean square gradient being the norm of the gradient times the square root of the number of (unfixed) atoms. This allowed a single low-energy conformation to be produced for each ligand. For each compound, the protonated conformation was taken into account based on the wash module implemented in MOE [54].

Concerning in silico protein preparation, the X-ray data of the α_2_-ADR receptor (PDB code = 6KUY) [50] and the AlphaFold model of *h*TAAR1 (AF-Q96RJ0-F1) [49] have been exploited. Both were managed thanks to the “Structure Preparation” tool from MOE 2019.01 suite. Afterward, the “Protonate3D” tool was exploited to assign the most probable protonation state to each residue while partial charges were attributed according to the AMBER10:EHT force field, as included in MOE.

### 3.2. Molecular Docking Studies

Molecular docking simulations at the α_2_-ADR receptor have been performed by means of the Dock module implemented in MOE software (2019.01 version), applying the template-based approach using the co-crystallized α_2_-ADR targeting ligand as a reference compound. As regards the *h*TAAR1 model, the corresponding ligand binding site has been determined based on superimposition to the α_2_-ADR protein, with the two GPCRs being aligned via Blosum62 (MOE software, 2019.01 version). This piece of information allowed the corresponding protein cavity to be derived at the superposed TAAR1 receptor and a preliminary docking run of the dual acting compound S18616 to be performed.

This was developed by applying the Alpha Triangle method and the Affinity ΔG prediction as the final scoring function. The obtained pose of the **S18616** ligand at the *h*TAAR1 receptor was then exploited as a reference compound for the following docking studies of the imidazole- and imidazoline-based derivatives at *h*TAAR1, via the template-based approach. In particular, the specific applied procedure was the same as described above. Details of the template-based docking calculations as well as of the scoring functions are shown in our previous papers.

### 3.3. QSAR Analyses

For the development of the quantitative–structure activity relationship (QSAR) models, any compound was explored in terms of geometry and conformation energy by means of the systematic conformational search tool implemented in MOE. For details, see our previous paper [30]. QSAR models A and B have been developed by using the *h*TAAR1 and α_2_-ADR binding affinity values as dependent variables while a set of molecular descriptors have been exploited as independent ones. The two final models have been derived applying the chemoinformatic and QSAR packages of MOE, including the molecular descriptors calculation. Afterwards, 302 molecular descriptors (2D and 3D) were computed by this software, and the resulting matrix was submitted to (QSAR) analyses. A final data matrix of 45 objects (compounds/molecules) and 302 rows (molecular descriptors) was obtained. The chemometric package PARVUS [74] was applied to handle such information, in particular for checking the constant predictors, splitting the data into training and test sets, and selecting the most informative molecular descriptors in order to develop two independent QSAR models for TAAR1 pKi and α_2_ pKi, as described in detail below [55].

First of all, the CHECK module implemented in PARVUS was used for checking the constancy of variables in 5 cancellation groups, and 283 molecular descriptors were retained after elimination of the constant predictors. All the derivatives herein explored have been divided in a training set, for models A and B generation, and in a test set, for the two models’ validation. In particular, the Kennard–Stone duplex design [56] was used in order to split the data into representative training and test sets; this algorithm was applied using the first 8 principal components of the autoscaled data, considering 85% of the total variance. Then, 31 molecules were selected for the training set, and 14 molecules were assigned to the test set (30% of the total molecules).

Iterative stepwise elimination PLS (ISEPLS) [75] was then applied as a variable selection method in order to evaluate the relevance of the selected predictors with regard to the possibility of predicting the two response variables (TAAR1 pKi and α_2_ pKi) independently. ISE is based on the importance the predictors, defined as (Equation (3)):

(3) $$z_{v}=\frac{\left|b_{v}\right|s_{v}}{\sum_{v=1}^{V}\left|b_{v}\right|s_{v}}$$

where b_v_ is the regression coefficient and s_v_ the standard deviation of the descriptor v. In each elimination cycle, the descriptor with the minimum importance is eliminated, and the model is computed again with the remaining predictors. The best model is selected on the basis of the predictive ability in cross validation. The two final models for TAAR1 pKi and α_2_ pKi retained 10 and 11 relevant descriptors, respectively.

More details about the selected descriptors are shown as follows: (i) Surface area, volume, and shape descriptors depend on the structure connectivity and conformation (dimensions are measured in Å), and the vsurf descriptors are similar to the VolSurf descriptors [76]. (ii) Subdivided surface areas are based on an approximate accessible van der Waals surface area (in Å^2^) calculation for each atom, vi along with some other atomic property, pi. The vi are calculated using a connection table approximation. Each descriptor in a series is defined to be the sum of the vi over all atoms i such that pi is in a specified range (a,b).

For the corresponding description, Li denotes the contribution to logP(o/w) for atom i as calculated in the SlogP descriptor [59]. Ri denotes the contribution to molar refractivity for atom i as calculated in the SMR descriptor [59]. The ranges were determined by the percentile subdivision over a large collection of compounds. (iii) Partial charge descriptors; depending on the partial charge of each atom of a chemical structure, require calculation of those partial charges. Partial charges from forcefields can be used by energy minimizing the database structures (which will store the charges) and then using the Q_ variant of the descriptors. Let qi denote the partial charge of atom i as defined above. Let vi be the van der Waals surface area (Å2) of atom i (as calculated by a connection table approximation). (iv) Conformation dependent charge descriptors depend upon the stored partial charges of the molecules and their conformations. Accessible surface area refers to the water accessible surface (in Å^2^) area using a probe radius of 1.4 Å. Let qi denote the partial charge of atom i. (v) Potential energy descriptors use the MOE potential energy model to calculate energetic quantities (in kcal/mol) from stored 3D conformations. (vi) Adjacency and distance matrix descriptors; being the adjacency matrix, M, of a chemical structure defined by the elements [Mij] where Mij is one if atoms i and j are bonded and zero otherwise. The distance matrix, D, of a chemical structure is defined by the elements [Dij] where Dij is the length of the shortest path from atoms i to j; zero is used if atoms i and j are not part of the same connected component. Petitjean [77] defines the eccentricity of a vertex to be the longest path from that vertex to any other vertex in the graph. The graph radius is the smallest vertex eccentricity in the graph, and the graph diameter as the largest vertex eccentricity. These values are calculated using the distance matrix and are used for several descriptors described below. (vii) Pharmacophore feature descriptors consider only the heavy atoms of a molecule and assign a type to each atom. That is, hydrogens are suppressed during the calculation. (viii) Subdivided surface areas, including descriptors based on an approximate accessible van der Waals surface area (in Å^2^) calculation for each atom, v_i_ along with some other atomic property, p_i_. The vi are calculated using a connection table approximation. Each descriptor in a series is defined to be the sum of the vi over all atoms i such that p_i_ is in a specified range (a,b).

Li denotes the contribution to logP(o/w) for atom i as calculated in the SlogP descriptor [59]. Ri denotes the contribution to molar refractivity for atom i as calculated in the SMR descriptor [59]. The ranges were determined by the percentile subdivision over a large collection of compounds. PLS regression was performed on these two reduced data matrices, and the results were obtained in terms of predictive ability of the biological data (TAAR1 pKi and α_2_ pKi). Regarding test set compounds, the predictive ability of the two models A and B was expressed as r^2^_pred_ by applying the following equation Equation (4):

r^2^_pred_ = (SD-PRESS)/SD (4)

where SD is the sum of the squared deviations between the biological activities of the test set molecules and the mean activity of the training set compounds, and PRESS is the sum of the squared deviation between the observed and the predicted activities of the test set compounds.

Then, the development of the two pharmacophore models PM_A and PM_B have been performed by means of the pharmacophore search module implemented in the MOE software (2019.01 version), starting from the alignment of the selected hTAAR1 agonists (pKi > 8 M) **12**, **23**-**26**, **30,** and **34**, and the α_2_-ADR ligands (pKi > 7.5 M), **5b**, **12**, **14**, **20**, and **33**, respectively. For each model, only the pharmacophore features (F) shared by all the selected compounds have been reported, as aromatic or hydrophobic groups (AroǀHyd) or only hydrophobic- Hyd) or aromatic-cores (Aro), or H-bonding acceptor (Acc) or donor (Don) groups.

Details of the pharmacophore search module have been previously reported by us [31]. This kind of analysis is described in the literature as a useful approach to guide the design of novel bioactive compounds [78,79,80].

### 3.4. Reagents

Guanfacine hydrochloride and guanabenz acetate are commercially available (Sigma-Aldrich, Milan, Italy or St. Louis, MO, USA). Cell culture reagents and buffers were from Gibco (Thermo Fisher Scientific, New York, NY, USA) or Invitrogen (Thermo Fisher Scientific, Carlsbad, CA, USA) and Sigma-Aldrich (Milan, Italy). Coelenterazine *h* was purchased from Promega (Milan, Italy). Plasmid containing the cDNA for *h*TAAR1 was a generous gift from Hoffman-La Roche.

### 3.5. Cell Culture and BRET Experiment

Human embryonic kidney 293 cells (HEK293T; ATCC CRL-11268) were maintained in Dulbecco’s Modified Eagle’s medium (DMEM; Gibco-Thermo Fisher Scientific, New York, NY, USA) supplemented with 10% (*v*/*v*) of FBS, 2 mM l-glutamine, and 0.05 mg/mL of gentamicin (both from Gibco) at 37 °C in a humidified atmosphere at 95% air and 5% CO_2_. Transient transfections were performed 24 h after cell seeding using the Lipofectamine reagent 2000 protocol (Invitrogen-Thermo Fisher Scientific, Carlsbad, USA). Then, 5 µg of *h*TAAR1 and 4 µg of cAMP biosensor protein (EPAC) encoding plasmids (the latter based on pcDNA3 core vector [59]) for each milliliter of transfection mix were used. For the BRET experiments, the cells were plated 6 h after transfection in poly-D-lysine coated 96-well white opaque microplates (Corning, New York, NY, USA) at a density of 7 × 10^4^ cells per well in phenol red free Minimum Essential Medium (MEM; Gibco-Thermo Fisher Scientific, New York, NY, USA) containing 2% of FBS, 10 mM HEPES buffering agent, and 2 mM L-glutamine (all from Gibco-Thermo Fisher Scientific, New York, NY, USA). The cells were then cultured for an additional 24 h.

The BRET experiment was conducted as already described [65]. Briefly, for time course experiments, the plate was read immediately after the addition of the agonist and for approximately 20 min. All the experiments were conducted in the presence of the phosphodiesterase inhibitor 3-isobutyl-1-methylxanthine (IBMX; Sigma-Aldrich, St. Louis, MO, USA) at the final concentration of 200 µM. Readings were collected using a Tecan Infinite instrument that allows the sequential integration of the signals detected in the 465 to 505 nm and 515 to 555 nm windows using filters with the appropriate band pass and by using iControl software. The acceptor/donor ratio was then calculated.

All the compounds were dissolved in dimethyl sulfoxide (DMSO; Sigma-Aldrich, St. Louis, MO, USA) and tested at the final concentration of 10 μM. Beta-phenylethylamine (Sigma-Aldrich, St. Louis, MO, USA) at the final concentration of 10 µM was applied as a positive control. To confirm specificity of positive responses, parallel screening on cells not transfected with the TAAR1-encoding vector was carried out. For compounds considered active, separate dose-response experiments were performed in order to calculate the EC_50_ values. Curves were fitted using a non-linear regression and one site specific binding with GraphPad Prism 6 (GraphPad Software). Data are representative of 4–5 independent experiments and are expressed as means, SD < 10%.

### 3.6. Subjects

Female rats with a loss-of-function mutation of the dopamine transporter gene (DAT-KO, n = 6) and their wild type (WT, n = 8) littermates were derived from the previously described rat strain [71]. One rat was excluded from the 3rd test because of illness-induced weight loss. All used animals were drug and test naïve before the start of experiments and were housed under a 12 h/12 h light/dark cycle (lights on at 08:00 h) at 21 ± 2 °C and 50 ± 20% humidity at the animal facility of Valdman Institute of pharmacology. Animals were housed in groups of siblings (3–5 per TIV (rats) or TIII cage (Tecniplast, Varese, Italy)). During the experiments, animals had free access to filtered (“AQUAPHOR”, Saint Petersburg, Russia) tap water and standard laboratory rat chow (receipt ΠK 120-1, KKZ “Laboratorkorm”, Moscow, Russia). The cages, corn cob bedding (“KKZ ‘Zolotoy pochatok’” LLC, Voronezh, Russia), and water bottles were changed once a week.

All tests were performed during the light period of the light/dark cycle. Experimental protocols were approved by the Local Animal Care and Use committee (First Pavlov State Saint Petersburg Medical University, #100_ИΦ1_012017/3_900 and #100_ИΦ1_012019/21_300).

### 3.7. Locomotor Activity Measurement

The study was performed in the apparatus ‘Actometer’ described in detail before [81]. We used the number of ambulation (sequential beam breaks) to estimate the horizontal locomotor activity of animals. We performed tests with guanfacine in the rats using a within-subjects design. Before guanfacine or vehicle injection (dose order based on Latin Square design), the rats were habituated to the activity monitor for 30 min. Following the injection, the locomotor activity of the animals was recorded for an additional 60 min divided in 12 intervals of 5-min each.

### 3.8. Statistical Analysis

Alpha was set at 0.05. All statistical analyses were performed using IBM SPSS Statistics 21 (IBM, Armonk, New York, NY, USA). We chose non-parametric methods to analyze results because tests for normality are not able to work properly in cases of small (n less than 40) sample sizes.

## 4. Conclusions

Molecular docking studies combined with QSAR analyses allowed us to investigate the main requirements turning in putative selective TAAR1 or α_2_-ADR ligands. In particular, the pivotal role showed by a proper folded conformation interacting with TAAR1 is confirmed by the high number of aromatic residues included in the *h*TAAR1 pocket, if compared to that of α_2_-ADR. This piece of information turns in preferred aromatic scaffolds for the TAAR1 agonism, as also confirmed by the QSAR studies. Indeed, most of the descriptors involved in model A (TAAR1 binding ability) rather than those of model B (α_2_-ADR binding ability) were clustered as conformer-dependent descriptors. Comparing the two models, *h*TAAR1 and α_2_-ADR binding affinity values increase in the presence of polar ligands (via DipoleY parameter) and positively charged moieties (Q_VSA_PNEG), respectively. On the other hand, the presence of an overall hydrophobic structure is in any case encouraged. The development of specific pharmacophore models (PM_A and PM_B) allowed us to interpret the main features to be included in future *h*TAAR1/α_2_-ADR ligands. On this qualitative information, the compound guanfacine was explored as the putative dual acting *h*TAAR1/α_2_-ADR ligand. Guanfacine was demonstrated to potently agonize the *h*TAAR1 receptor at the same rank of α_2_-ADR, as also observed for the reference drug guanabenz. From a biological standpoint, the here disclosed dual agonism activity of guanfacine could represent a suitable tool for deepening the pharmacology of TAAR1 and its fine interconnection with the α_2_-adrenergic system; from the medicinal chemistry point of view, guanfacine arises as an interesting template molecule for further structural variations with an attempt to develop selective TAAR1 agonists. Despite this centrally active α_2_-ADR drug being known for over 50 years, a better understanding of its biological multifunctional profile and potential application in novel therapeutic areas remains an intriguing matter, necessitating subsequent investigation.

## Figures and Tables

**Table 1:** Comparison of the *h*TAAR and α_2_-ADR as based on the alignment of the two proteins (Figure S3). Residues 3.5 Å far from the 6KUY co-crystallized ligand are listed.

Protein	Corresponding Residues				
**6KUY**	Asp113	Val114	Cys117	Ile190	Ser204
***h*TAAR1**	Asp103	Ile104	Ser107	Val184	Ser198
**6KUY**	Trp387	Phe390	Phe391	Phe412	Tyr416
***h*TAAR1**	Trp264	Phe267	Phe268	Ile290	Tyr294

**Table 2:** List of the selected descriptors in tandem with the related type, series, and relative importance index (RI). Negatively related descriptors are shown in italics.

Molecular Descriptor Code	Descriptor Class	Description	Descriptor Type	Relative Importance (RI)
DipoleY	3D-V	The y component of the dipole moment (external coordinates).	Conformation Dependent Charge Descriptors	1.00000
SlogP_VSA4	2D-II	Sum of vi such that Li is in (0.1, 0.15].	Subdivided Surface Areas	0.810513
*DCASA*	3D-V	Absolute value of the difference between CASA+ (Positive charge weighted surface area, ASA+ times max {qi > 0}) and CASA- (Negative charge weighted surface area, ASA- times max {qi < 0}). [58]	Conformation Dependent Charge Descriptors	0.582248
*E*	3D-I	Value of the potential energy. The state of all term enable flags will be honored (in addition to the term weights). This means that the current potential setup accurately reflects what will be calculated.	Potential Energy Descriptors	0.435867
vsurf_IW5	3D-III	Hydrophilic integy moment (8 descriptors)	Surface Area, Volume and Shape Descriptors	0.344589
DipoleZ	3D-V	The z component of the dipole moment (external coordinates).	Conformation Dependent Charge Descriptors	0.325382
*Q_VSA_FHYD*	2D-V	Fractional hydrophobic van der Waals surface area. This is the sum of the vi such that |qi| is less than or equal to 0.2 divided by the total surface area. The vi are calculated using a connection table approximation.	Partial Charge Descriptors	0.31853
*vsurf_EDmin1*	3D-III	Lowest hydrophobic energy (3 descriptors)	Surface Area, Volume and Shape Descriptors	0.29631
*Q_RPC-*	2D-V	Relative negative partial charge: the smallest negative qi divided by the sum of the negative qi. Q_RPC- is identical to RPC- which has been retained for compatibility.	Partial Charge Descriptors	0.061043
*GCUT_SMR_0*	2D-VII	The GCUT descriptors using atomic contribution to molar refractivity (using the Wildman and Crippen SMR method) [59] instead of partial charge.	Adjacency and Distance Matrix Descriptors	0.027644

**Table 3:** List of the selected descriptors, in tandem with the related type, series, and relative importance index (RI). Negatively related descriptors are shown in italics.

Molecular Descriptor Code	Descriptor Class	Description	Descriptor Type	Relative Importance (RI)
*Q_VSA_PNEG*	2D-V	Fractional negative van der Waals surface area. This is the sum of the vi such that qi is negative divided by the total surface area. The vi are calculated using a connection table approximation.	Partial Charge Descriptors	1.000000
Q_VSA_POL	2D-V	Total positive van der Waals surface area. This is the sum of the vi such that qi is non-negative. The vi are calculated using a connection table approximation.	Partial Charge Descriptors	0.795835
SlogP_VSA3	2D-II	Sum of vi such that Li is in (0,0.1].	Subdivided Surface Areas	0.195358
vsurf_ID7	3D-III	Hydrophobic integy moment (8 descriptors)	Surface Area, Volume and Shape Descriptors	0.156047
vsa_other	2D-VI	Approximation to the sum of VDW surface areas (Å2) of atoms typed as “other”.	Pharmacophore Feature Descriptors	0.102077
vsurf_IW4	3D-III	Hydrophilic integy moment (8 descriptors)	Surface Area, Volume and Shape Descriptors	0.1005
*E_tor*	3D-I	Torsion (proper and improper) potential energy. In the Potential Setup panel, the term enable (Bonded) flag is ignored, but the term weight is applied.	Potential Energy Descriptors	0.098422
*balabanJ*	2D-VII	Balaban’s connectivity topological index [60].	Adjacency and Distance Matrix Descriptors	0.085904
vsurf_ID1	3D-III	Hydrophobic integy moment (8 descriptors)	Surface Area, Volume and Shape Descriptors	0.022599
GCUT_SMR_1	2D-VII	The GCUT descriptors using atomic contribution to molar refractivity (using the Wildman and Crippen SMR method) instead of partial charge.	Adjacency and Distance Matrix Descriptors	0.005108
Q_VSA_FHYD	2D-V	Fractional hydrophobic van der Waals surface area. This is the sum of the vi such that |qi| is less than or equal to 0.2 divided by the total surface area. The vi are calculated using a connection table approximation.	Partial Charge Descriptors	0.001779

## Data Availability

Data is contained within the article.

## Supplementary Materials

The following supporting information can be downloaded at: https://www.mdpi.com/article/10.3390/ph16111632/s1, Figure S1. Pairwise Percentage Residue Identity (PPRI) and Similarity values as obtained by the *h*TAAR1 (shown as green ribbon) and α_2_-ADR (shown as gold ribbon) superposition. Figure S2. Superimposition of the hTAAR1 model and of the X-ray data about α_2_-ADR (left); the corresponding RMSD values are also reported (right). Figure S3. Alignment of the *h*TAAR1 and α_2_-ADR protein sequence. Figure S4. Full-view of the dual *h*TAAR1 (green) and α_2_-ADR (cyan) agonist **S18616** at the whole proteins. Ligand volume is highlighted in light brown. Figure S5. Docking positioning of **37** (C atom; magenta) at the *h*TAAR1 binding site. The most important residues involved in the agonist binding are labelled. Figure S6. Docking positioning of **53** (C atom; magenta) at the *h*TAAR1 binding site (A) and of **51** at the α_2_-ADR cavity (B). The most important residues involved in the agonist binding are labelled. Figure S7. Schematic representation of the opposite role played by the Q_VSA_FHYD descriptor, shared by both models A and B, to influence the compound (*h*TAAR1 and a_2_-ADR) binding affinity values. Compounds are represented as dots. Table S1. Five top scored docking positioning of **5a**-**5e**, **6a**-**6e**, **11**-**54** and the reference agonist **S18616** at the *h*TAAR1 (MOE software). The predicted ΔG value of each protein-ligand complex has been reported, as calculated in terms of final scoring function (S, as Kcal/mol). Table S2. Five top scored docking positioning of **5a**-**5e**, **6a**-**6e**, **11**-**54** and the reference agonist **S18616** at the α_2_-ADR (MOE software). The predicted ΔG value of each protein-ligand complex has been reported, as calculated in terms of final scoring function (S, as Kcal/mol). Table S3. Chemical structure of the dual acting *h*TAAR1 and α_2_ADR ligands. The corresponding binding affinity values are also reported. Table S4. The predicted (Pred. pKi) and experimental (Exp. pKi) *h*TAAR1 binding affinity values of the compounds (Comp.) herein explored are reported, in tandem with the collected descriptors. The compounds included in the test set are underlined along the first column. Table S5. The predicted (Pred. pKi) and experimental (Exp. pKi) α_2_-ADR binding affinity values of the compounds (Comp.) herein explored are reported, in tandem with the collected descriptors. The compounds included in the test set are underlined along the first column.

## References

[1] Rutigliano G., Accorroni A., Zucchi R. (2017). The Case for TAAR1 as a Modulator of Central Nervous System Function. Front. Pharmacol..

[2] Barnes D.A., Hoener M.C., Moore C.S., Berry M.D. (2023). TAAR1 Regulates Purinergic-Induced TNF Secretion from Peripheral, But Not CNS-Resident, Macrophages. J. Neuroimmune Pharmacol..

[3] Halff E.F., Rutigliano G., Garcia-Hidalgo A., Howes O.D. (2023). Trace Amine-Associated Receptor 1 (TAAR1) Agonism as a New Treatment Strategy for Schizophrenia and Related Disorders. Trends Neurosci..

[4] Zhai L., Xiao H., Lin C., Wong H.L.X., Lam Y.Y., Gong M., Wu G., Ning Z., Huang C., Zhang Y. (2023). Gut Microbiota-Derived Tryptamine and Phenethylamine Impair Insulin Sensitivity in Metabolic Syndrome and Irritable Bowel Syndrome. Nat. Commun..

[5] Liu H., Zheng Y., Wang Y., Wang Y., He X., Xu P., Huang S., Yuan Q., Zhang X., Wang L. (2023). Recognition of Methamphetamine and Other Amines by Trace Amine Receptor TAAR1. Nature.

[6] Wu R., Li J.-X. (2021). Potential of Ligands for Trace Amine-Associated Receptor 1 (TAAR1) in the Management of Substance Use Disorders. CNS Drugs.

[7] Alnefeesi Y., Tamura J.K., Lui L.M.W., Jawad M.Y., Ceban F., Ling S., Nasri F., Rosenblat J.D., McIntyre R.S. (2021). Trace Amine-Associated Receptor 1 (TAAR1): Potential Application in Mood Disorders: A Systematic Review. Neurosci. Biobehav. Rev..

[8] Raony Í., Domith I., Lourenco M.V., Paes-de-Carvalho R., Pandolfo P. (2022). Trace Amine-Associated Receptor 1 Modulates Motor Hyperactivity, Cognition, and Anxiety-like Behavior in an Animal Model of ADHD. Prog. Neuro-Psychopharmacol. Biol. Psychiatry.

[9] Sakanoue W., Yokoyama T., Hirakawa M., Maesawa S., Sato K., Saino T. (2023). 3-Iodothyronamine, a Trace Amine-Associated Receptor Agonist, Regulates Intracellular Ca2+ Increases via CaMK II through Epac2 in Rat Cerebral Arterioles. Biomed. Res..

[10] Decker A.M., Brackeen M.F., Mohammadkhani A., Kormos C.M., Hesk D., Borgland S.L., Blough B.E. (2022). Identification of a Potent Human Trace Amine-Associated Receptor 1 Antagonist. ACS Chem. Neurosci..

[11] Bugda Gwilt K., González D.P., Olliffe N., Oller H., Hoffing R., Puzan M., El Aidy S., Miller G.M. (2020). Actions of Trace Amines in the Brain-Gut-Microbiome Axis via Trace Amine-Associated Receptor-1 (TAAR1). Cell. Mol. Neurobiol..

[12] Perini F., Nazimek J.M., Mckie S., Capitão L.P., Scaife J., Pal D., Browning M., Dawson G.R., Nishikawa H., Campbell U. (2023). Effects of Ulotaront on Brain Circuits of Reward, Working Memory, and Emotion Processing in Healthy Volunteers with High or Low Schizotypy. Schizophrenia.

[13] Ågren R., Betari N., Saarinen M., Zeberg H., Svenningsson P., Sahlholm K. (2023). In Vitro Comparison of Ulotaront (SEP-363856) and Ralmitaront (RO6889450): Two TAAR1 Agonist Candidate Antipsychotics. Int. J. Neuropsychopharmacol..

[14] Tonelli M., Cichero E. (2020). Trace Amine Associated Receptor 1 (TAAR1) Modulators: A Patent Review (2010-Present). Expert. Opin. Ther. Pat..

[15] Frycz B.A., Nowicka K., Konopka A., Hoener M.C., Bulska E., Kaczmarek L., Stefaniuk M. (2023). Activation of Trace Amine-Associated Receptor 1 (TAAR1) Transiently Reduces Alcohol Drinking in Socially Housed Mice. Addict. Biol..

[16] Grinchii D., Hoener M.C., Khoury T., Dekhtiarenko R., Nejati Bervanlou R., Jezova D., Dremencov E. (2022). Effects of Acute and Chronic Administration of Trace Amine-Associated Receptor 1 (TAAR1) Ligands on in Vivo Excitability of Central Monoamine-Secreting Neurons in Rats. Mol. Psychiatry.

[17] Dorotenko A., Tur M., Dolgorukova A., Bortnikov N., Belozertseva I.V., Zvartau E.E., Gainetdinov R.R., Sukhanov I. (2020). The Action of TAAR1 Agonist RO5263397 on Executive Functions in Rats. Cell. Mol. Neurobiol..

[18] Wu R., Liu J., Wang K., Huang Y., Zhang Y., Li J.-X. (2020). Effects of a Trace Amine-Associated Receptor 1 Agonist RO 5263397 on Ethanol-Induced Behavioral Sensitization. Behav. Brain Res..

[19] Polini B., Ricardi C., Bertolini A., Carnicelli V., Rutigliano G., Saponaro F., Zucchi R., Chiellini G. (2023). T1AM/TAAR1 System Reduces Inflammatory Response and β-Amyloid Toxicity in Human Microglial HMC3 Cell Line. Int. J. Mol. Sci..

[20] Kane J.M. (2022). A New Treatment Paradigm: Targeting Trace Amine-Associated Receptor 1 (TAAR1) in Schizophrenia. J. Clin. Psychopharmacol..

[21] Dedic N., Dworak H., Zeni C., Rutigliano G., Howes O.D. (2021). Therapeutic Potential of TAAR1 Agonists in Schizophrenia: Evidence from Preclinical Models and Clinical Studies. Int. J. Mol. Sci..

[22] Kantrowitz J.T. (2021). Trace Amine-Associated Receptor 1 as a Target for the Development of New Antipsychotics: Current Status of Research and Future Directions. CNS Drugs.

[23] Dedic N., Jones P.G., Hopkins S.C., Lew R., Shao L., Campbell J.E., Spear K.L., Large T.H., Campbell U.C., Hanania T. (2019). SEP-363856, a Novel Psychotropic Agent with a Unique, Non-D2 Receptor Mechanism of Action. J. Pharmacol. Exp. Ther..

[24] Dodd S., Carvalho A.F., Puri B.K., Maes M., Bortolasci C.C., Morris G., Berk M. (2021). Trace Amine-Associated Receptor 1 (TAAR1): A New Drug Target for Psychiatry?. Neurosci. Biobehav. Rev..

[25] Achtyes E.D., Hopkins S.C., Dedic N., Dworak H., Zeni C., Koblan K. (2023). Ulotaront: Review of Preliminary Evidence for the Efficacy and Safety of a TAAR1 Agonist in Schizophrenia. Eur. Arch. Psychiatry Clin. Neurosci..

[26] Koblan K.S., Kent J., Hopkins S.C., Krystal J.H., Cheng H., Goldman R., Loebel A. (2020). A Non-D2-Receptor-Binding Drug for the Treatment of Schizophrenia. N. Engl. J. Med..

[27] Højlund M., Correll C.U. (2022). Ulotaront: A TAAR1/5-HT1A Agonist in Clinical Development for the Treatment of Schizophrenia. Expert Opin. Investig. Drugs.

[28] Hart M.E., Suchland K.L., Miyakawa M., Bunzow J.R., Grandy D.K., Scanlan T.S. (2006). Trace Amine-Associated Receptor Agonists: Synthesis and Evaluation of Thyronamines and Related Analogues. J. Med. Chem..

[29] Tonelli M., Espinoza S., Gainetdinov R.R., Cichero E. (2017). Novel Biguanide-Based Derivatives Scouted as TAAR1 Agonists: Synthesis, Biological Evaluation, ADME Prediction and Molecular Docking Studies. Eur. J. Med. Chem..

[30] Guariento S., Tonelli M., Espinoza S., Gerasimov A.S., Gainetdinov R.R., Cichero E. (2018). Rational Design, Chemical Synthesis and Biological Evaluation of Novel Biguanides Exploring Species-Specificity Responsiveness of TAAR1 Agonists. Eur. J. Med. Chem..

[31] Francesconi V., Cichero E., Kanov E.V., Laurini E., Pricl S., Gainetdinov R.R., Tonelli M. (2020). Novel 1-Amidino-4-Phenylpiperazines as Potent Agonists at Human TAAR1 Receptor: Rational Design, Synthesis, Biological Evaluation and Molecular Docking Studies. Pharmaceuticals.

[32] Cichero E., Tonelli M. (2017). Targeting Species-Specific Trace Amine-Associated Receptor 1 Ligands: To Date Perspective of the Rational Drug Design Process. Future Med. Chem..

[33] Xu Z., Li Q. (2020). TAAR Agonists. Cell. Mol. Neurobiol..

[34] Millan M.J., Dekeyne A., Newman-Tancredi A., Cussac D., Audinot V., Milligan G., Duqueyroix D., Girardon S., Mullot J., Boutin J.A. (2000). S18616, a Highly Potent, Spiroimidazoline Agonist at Alpha(2)-Adrenoceptors: I. Receptor Profile, Antinociceptive and Hypothermic Actions in Comparison with Dexmedetomidine and Clonidine. J. Pharmacol. Exp. Ther..

[35] Galley G., Stalder H., Goergler A., Hoener M.C., Norcross R.D. (2012). Optimisation of Imidazole Compounds as Selective TAAR1 Agonists: Discovery of RO5073012. Bioorganic Med. Chem. Lett..

[36] Galley G., Beurier A., Décoret G., Goergler A., Hutter R., Mohr S., Pähler A., Schmid P., Türck D., Unger R. (2016). Discovery and Characterization of 2-Aminooxazolines as Highly Potent, Selective, and Orally Active TAAR1 Agonists. ACS Med. Chem. Lett..

[37] Childress A., Hoo-Cardiel A., Lang P. (2020). Evaluation of the Current Data on Guanfacine Extended Release for the Treatment of ADHD in Children and Adolescents. Expert Opin. Pharmacother..

[38] Ota T., Yamamuro K., Okazaki K., Kishimoto T. (2021). Evaluating Guanfacine Hydrochloride in the Treatment of Attention Deficit Hyperactivity Disorder (ADHD) in Adult Patients: Design, Development and Place in Therapy. Drug Des. Dev. Ther..

[39] Galvez-Contreras A.Y., Vargas-de la Cruz I., Beltran-Navarro B., Gonzalez-Castaneda R.E., Gonzalez-Perez O. (2022). Therapeutic Approaches for ADHD by Developmental Stage and Clinical Presentation. Int. J. Environ. Res. Public Health.

[40] Rizzo R., Martino D. (2015). Guanfacine for the Treatment of Attention Deficit Hyperactivity Disorder in Children and Adolescents. Expert Rev. Neurother..

[41] Black B.T., Soden S.E., Kearns G.L., Jones B.L. (2016). Clinical and Pharmacologic Considerations for Guanfacine Use in Very Young Children. J. Child Adolesc. Psychopharmacol..

[42] Alamo C., López-Muñoz F., Sánchez-García J. (2016). Mechanism of Action of Guanfacine: A Postsynaptic Differential Approach to the Treatment of Attention Deficit Hyperactivity Disorder (Adhd). Actas Esp. Psiquiatr..

[43] Ramos B.P., Arnsten A.F.T. (2007). Adrenergic Pharmacology and Cognition: Focus on the Prefrontal Cortex. Pharmacol. Ther..

[44] Arnsten A.F.T., Jin L.E. (2012). Guanfacine for the Treatment of Cognitive Disorders: A Century of Discoveries at Yale. Yale J. Biol. Med..

[45] Arnsten A.F.T. (2020). Guanfacine’s Mechanism of Action in Treating Prefrontal Cortical Disorders: Successful Translation across Species. Neurobiol. Learn. Mem..

[46] Fox H., Sofuoglu M., Sinha R. (2015). Guanfacine Enhances Inhibitory Control and Attentional Shifting in Early Abstinent Cocaine-Dependent Individuals. J. Psychopharmacol..

[47] Franowicz J.S., Kessler L.E., Borja C.M.D., Kobilka B.K., Limbird L.E., Arnsten A.F.T. (2002). Mutation of the alpha2A-Adrenoceptor Impairs Working Memory Performance and Annuls Cognitive Enhancement by Guanfacine. J. Neurosci..

[48] Jumper J., Evans R., Pritzel A., Green T., Figurnov M., Ronneberger O., Tunyasuvunakool K., Bates R., Žídek A., Potapenko A. (2021). Highly Accurate Protein Structure Prediction with AlphaFold. Nature.

[49] Varadi M., Anyango S., Deshpande M., Nair S., Natassia C., Yordanova G., Yuan D., Stroe O., Wood G., Laydon A. (2022). AlphaFold Protein Structure Database: Massively Expanding the Structural Coverage of Protein-Sequence Space with High-Accuracy Models. Nucleic Acids Res..

[50] Qu L., Zhou Q.T., Wu D., Zhao S.W. Crystal Structure of the alpha2A Adrenergic Receptor in Complex with a Partial Agonist. https://www.rcsb.org/structure/6KUY.

[51] Pettersen E.F., Goddard T.D., Huang C.C., Couch G.S., Greenblatt D.M., Meng E.C., Ferrin T.E. (2004). UCSF Chimera--a Visualization System for Exploratory Research and Analysis. J. Comput. Chem..

[52] The PyMOL Molecular Graphics System (2020).

[53] Wang Y.-L., Li J.-Y., Shi X.-X., Wang Z., Hao G.-F., Yang G.-F. (2021). Web-Based Quantitative Structure—Activity Relationship Resources Facilitate Effective Drug Discovery. Top. Curr. Chem..

[54] (2021). Molecular Operating Environment (MOE).

[55] Righetti G., Casale M., Liessi N., Tasso B., Salis A., Tonelli M., Millo E., Pedemonte N., Fossa P., Cichero E. (2020). Molecular Docking and QSAR Studies as Computational Tools Exploring the Rescue Ability of F508del CFTR Correctors. Int. J. Mol. Sci..

[56] Kennard R.W., Stone L.A. (1969). Computer Aided Design of Experiments. Technometrics.

[57] Forina M., Boggia R., Mosti L., Fossa P. (1997). Zupan’s Descriptors in QSAR Applied to the Study of a New Class of Cardiotonic Agents. Farmaco.

[58] Stanton D.T., Jurs P.C. (1990). Development and Use of Charged Partial Surface Area Structural Descriptors in Computer-Assisted Quantitative Structure-Property Relationship Studies. Anal. Chem..

[59] Wildman S.A., Crippen G.M. (1999). Prediction of Physicochemical Parameters by Atomic Contributions. J. Chem. Inf. Comput. Sci..

[60] Balaban A.T. (1982). Highly Discriminating Distance-Based Topological Index. Chem. Phys. Lett..

[61] Moro S., Bacilieri M., Cacciari B., Bolcato C., Cusan C., Pastorin G., Klotz K.-N., Spalluto G. (2006). The Application of a 3D-QSAR (autoMEP/PLS) Approach as an Efficient Pharmacodynamic-Driven Filtering Method for Small-Sized Virtual Library: Application to a Lead Optimization of a Human A3 Adenosine Receptor Antagonist. Bioorganic Med. Chem..

[62] Dudek M., Knutelska J., Bednarski M., Nowiński L., Zygmunt M., Mordyl B., Głuch-Lutwin M., Kazek G., Sapa J., Pytka K. (2015). A Comparison of the Anorectic Effect and Safety of the Alpha2-Adrenoceptor Ligands Guanfacine and Yohimbine in Rats with Diet-Induced Obesity. PLoS ONE.

[63] Kotańska M., Marcinkowska M., Kuder K.J., Walczak M., Bednarski M., Siwek A., Kołaczkowski M. (2023). Metabolic and Cardiovascular Benefits and Risks of 4-Hydroxy Guanabenz Hydrochloride: A2-Adrenoceptor and Trace Amine-Associated Receptor 1 Ligand. Pharmacol. Rep..

[64] Lam V.M., Rodríguez D., Zhang T., Koh E.J., Carlsson J., Salahpour A. (2015). Discovery of Trace Amine-Associated Receptor 1 Ligands by Molecular Docking Screening against a Homology Model. MedChemComm.

[65] Espinoza S., Masri B., Salahpour A., Gainetdinov R.R. (2013). BRET Approaches to Characterize Dopamine and TAAR1 Receptor Pharmacology and Signaling. Methods Mol. Biol..

[66] Hu L.A., Zhou T., Ahn J., Wang S., Zhou J., Hu Y., Liu Q. (2009). Human and Mouse Trace Amine-Associated Receptor 1 Have Distinct Pharmacology towards Endogenous Monoamines and Imidazoline Receptor Ligands. Biochem. J..

[67] Yoshino S., Iwasaki Y., Matsumoto S., Satoh T., Ozawa A., Yamada E., Kakizaki S., Trejo J.A.O., Uchiyama Y., Yamada M. (2020). Administration of Small-Molecule Guanabenz Acetate Attenuates Fatty Liver and Hyperglycemia Associated with Obesity. Sci. Rep..

[68] Kotańska M., Knutelska J., Nicosia N., Mika K., Szafarz M. (2022). Guanabenz-an Old Drug with a Potential to Decrease Obesity. Naunyn-Schmiedeberg–s Arch. Pharmacol..

[69] Michael E.S., Covic L., Kuliopulos A. (2019). Trace Amine-Associated Receptor 1 (TAAR1) Promotes Anti-Diabetic Signaling in Insulin-Secreting Cells. J. Biol. Chem..

[70] Raab S., Wang H., Uhles S., Cole N., Alvarez-Sanchez R., Künnecke B., Ullmer C., Matile H., Bedoucha M., Norcross R.D. (2016). Incretin-like Effects of Small Molecule Trace Amine-Associated Receptor 1 Agonists. Mol. Metab..

[71] Leo D., Sukhanov I., Zoratto F., Illiano P., Caffino L., Sanna F., Messa G., Emanuele M., Esposito A., Dorofeikova M. (2018). Pronounced Hyperactivity, Cognitive Dysfunctions, and BDNF Dysregulation in Dopamine Transporter Knock-out Rats. J. Neurosci..

[72] Savchenko A., Targa G., Fesenko Z., Leo D., Gainetdinov R.R., Sukhanov I. (2023). Dopamine Transporter Deficient Rodents: Perspectives and Limitations for Neuroscience. Biomolecules.

[73] Kurzina N., Belskaya A., Gromova A., Ignashchenkova A., Gainetdinov R.R., Volnova A. (2022). Modulation of Spatial Memory Deficit and Hyperactivity in Dopamine Transporter Knockout Rats via α2A-Adrenoceptors. Front. Psychiatry.

[74] V-PARVUS 2010. An Extendable Package of Programs for Explorative Data Analysis, Classification and Regression Analysis. Dip Chimica e Tecnologie Farmaceutiche, University of Genova. https://iris.unige.it/handle/11567/242687.

[75] Boggia R., Forina M., Fossa P., Mosti L. (1997). Chemometric Study and Validation Strategies in the Structure-Activity Relationships of New Cardiotonic Agents. Quant. Struct. Act. Relat..

[76] Cruciani G., Crivori P., Carrupt P.-A., Testa B. (2000). Molecular Fields in Quantitative Structure–Permeation Relationships: The VolSurf Approach. J. Mol. Struct. THEOCHEM.

[77] Petitjean M. (1992). Applications of the Radius-Diameter Diagram to the Classification of Topological and Geometrical Shapes of Chemical Compounds. J. Chem. Inf. Comput. Sci..

[78] Wolber G., Seidel T., Bendix F., Langer T. (2008). Molecule-Pharmacophore Superpositioning and Pattern Matching in Computational Drug Design. Drug Discov. Today.

[79] Khalid S., Hanif R., Jabeen I., Mansoor Q., Ismail M. (2018). Pharmacophore Modeling for Identification of Anti-IGF-1R Drugs and in-Vitro Validation of Fulvestrant as a Potential Inhibitor. PLoS ONE.

[80] Haidar S., Bouaziz Z., Marminon C., Laitinen T., Poso A., Le Borgne M., Jose J. (2017). Development of Pharmacophore Model for Indeno[1,2-b]Indoles as Human Protein Kinase CK2 Inhibitors and Database Mining. Pharmaceuticals.

[81] Sukhanov I., Dorofeikova M., Dolgorukova A., Dorotenko A., Gainetdinov R.R. (2018). Trace Amine-Associated Receptor 1 Modulates the Locomotor and Sensitization Effects of Nicotine. Front. Pharmacol..

