# Supplementary material for: Discovery of Guanfacine as a Novel TAAR1 Agonist: A Combination Strategy through Molecular Modeling Studies and Biological Assays
Source: Pharmaceuticals (Basel). 2023 Nov 20;16(11):1632. doi: 10.3390/ph16111632 (PMC10674299; doi:10.3390/ph16111632)
Supplement: Supplementary file 1 [file pharmaceuticals-16-01632-s001.zip › pharmaceuticals-2692752-supplementary.pdf]

SUPPORTING INFORMATION

Discovery of Guanfacine as a novel TAAR1 agonist: a combination strategy through molecular modeling studies and biological assays

Elena Cichero[1], Valeria Francesconi[1], Beatrice Casini[1], Monica Casale[2], Evgeny Kanov[3], Andrey S. Gerasimov[3], Ilya Sukhanov[4], Artem Savchenko[4], Stefano Espinoza[5,6], Raul R. Gainetdinov[3,7], Michele Tonelli[1]*

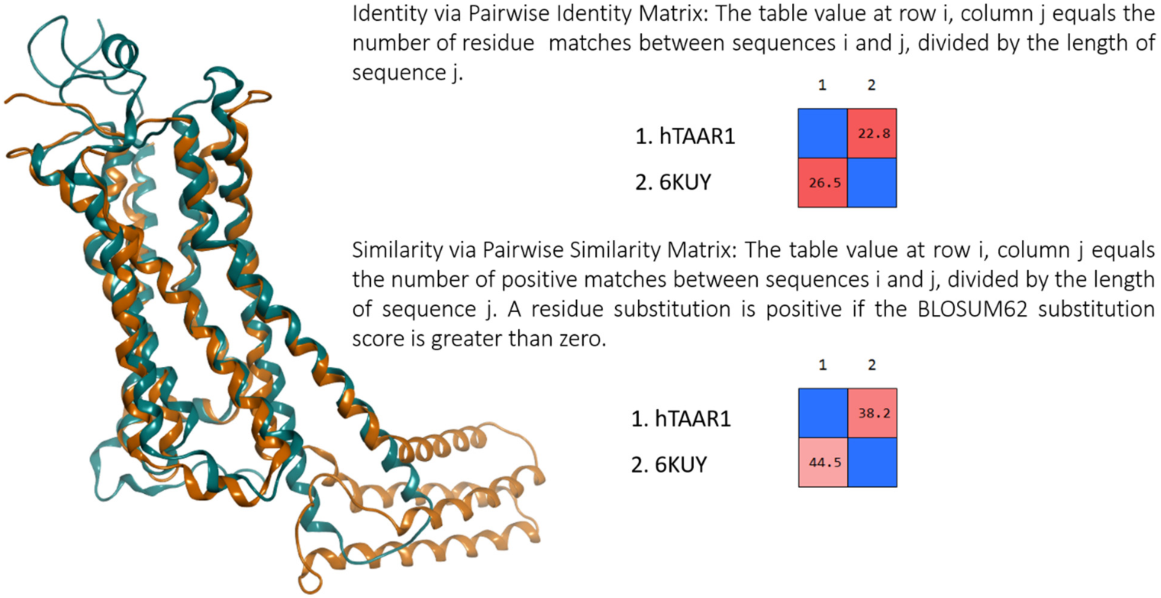

Identity via Pairwise Identity Matrix: The table value at row i, column j equals the number of residue matches between sequences i and j, divided by the length of sequence j.

	1	2
1. hTAAR1		22.8
2. 6KUY	26.5	

Similarity via Pairwise Similarity Matrix: The table value at row i, column j equals the number of positive matches between sequences i and j, divided by the length of sequence j. A residue substitution is positive if the BLOSUM62 substitution score is greater than zero.

	1	2
1. hTAAR1		38.2
2. 6KUY	44.5	

Figure S1. Pairwise Percentage Residue Identity (PPRI) and Similarity values as obtained by the *h*TAAR1 (shown as green ribbon) and α_2-ADR (shown as gold ribbon) superposition.

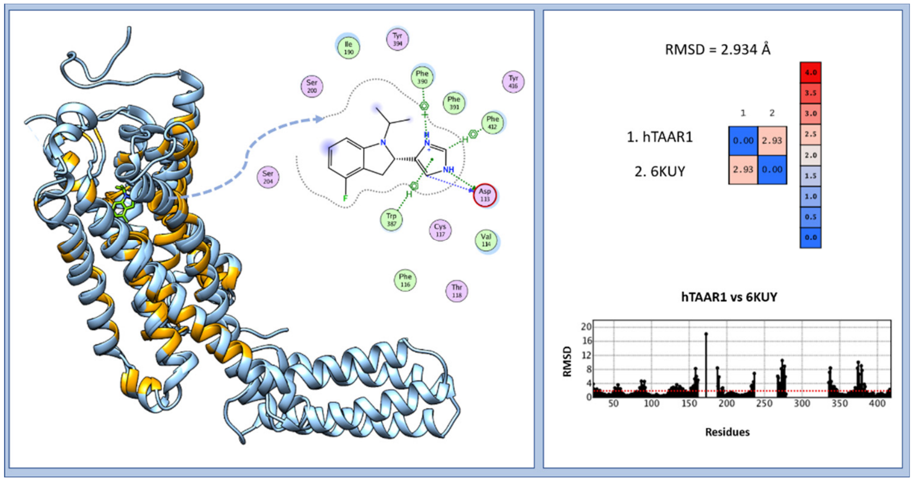

Figure S2. Superimposition of the hTAAR1 model and of the X-ray data about α2-ADR (left); the corresponding RMSD values are also reported (right).

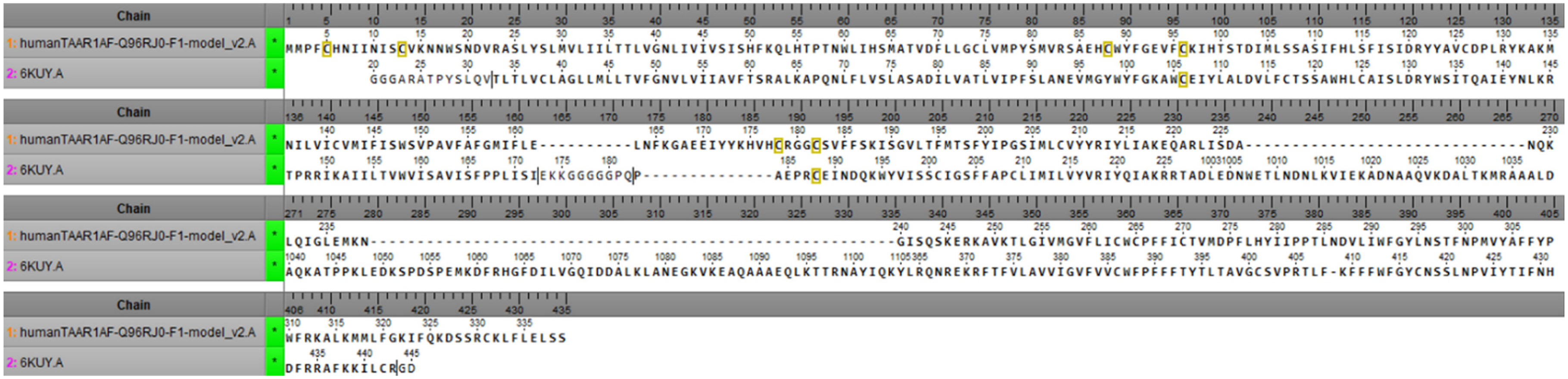

Figure S3. Alignment of the *h*TAAR1 and α2-ADR protein sequence.

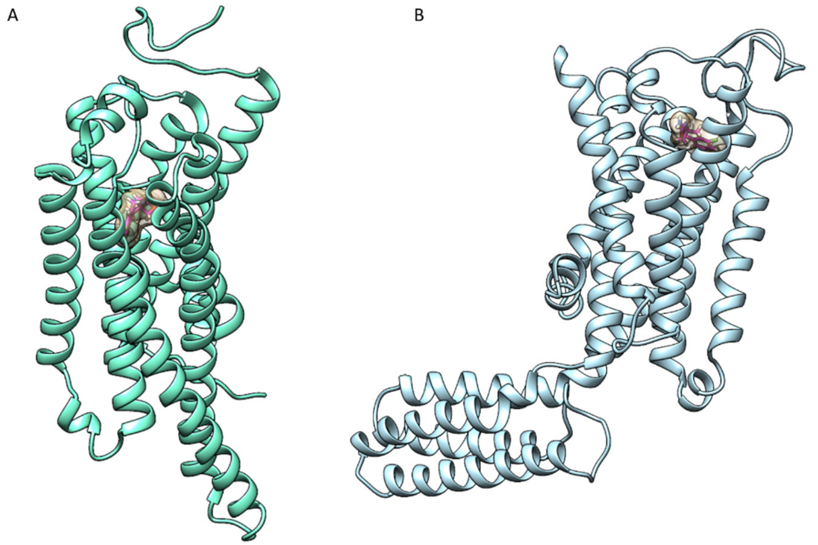

Figure S4. Full-view of the dual *h*TAAR1 (green) and α_2-ADR (cyan) agonist **S18616** at the whole proteins. Ligand volume is highlighted in light brown.

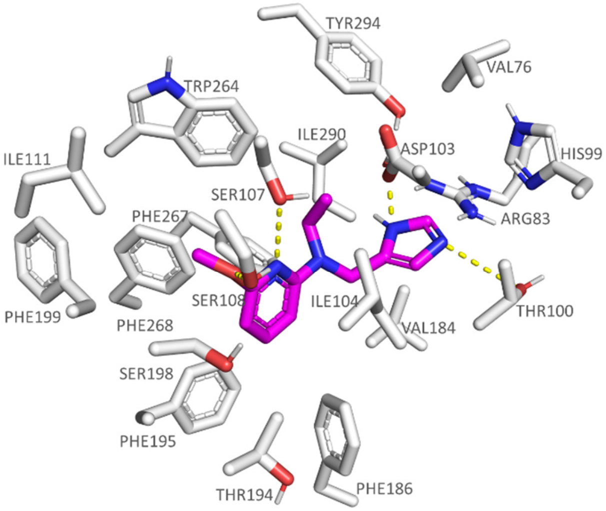

Figure S5. Docking positioning of **37** (C atom; magenta) at the *h*TAAR1 binding site. The most important residues involved in the agonist binding are labelled.

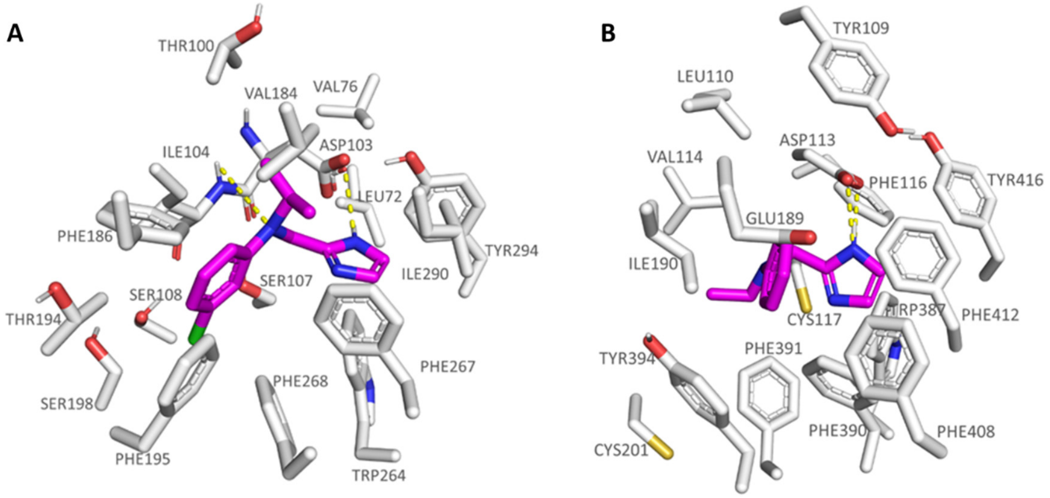

Figure S6. Docking positioning of **53** (C atom; magenta) at the *h*TAAR1 binding site (A) and of **51** at the α_2-ADR cavity (B). The most important residues involved in the agonist binding are labelled.

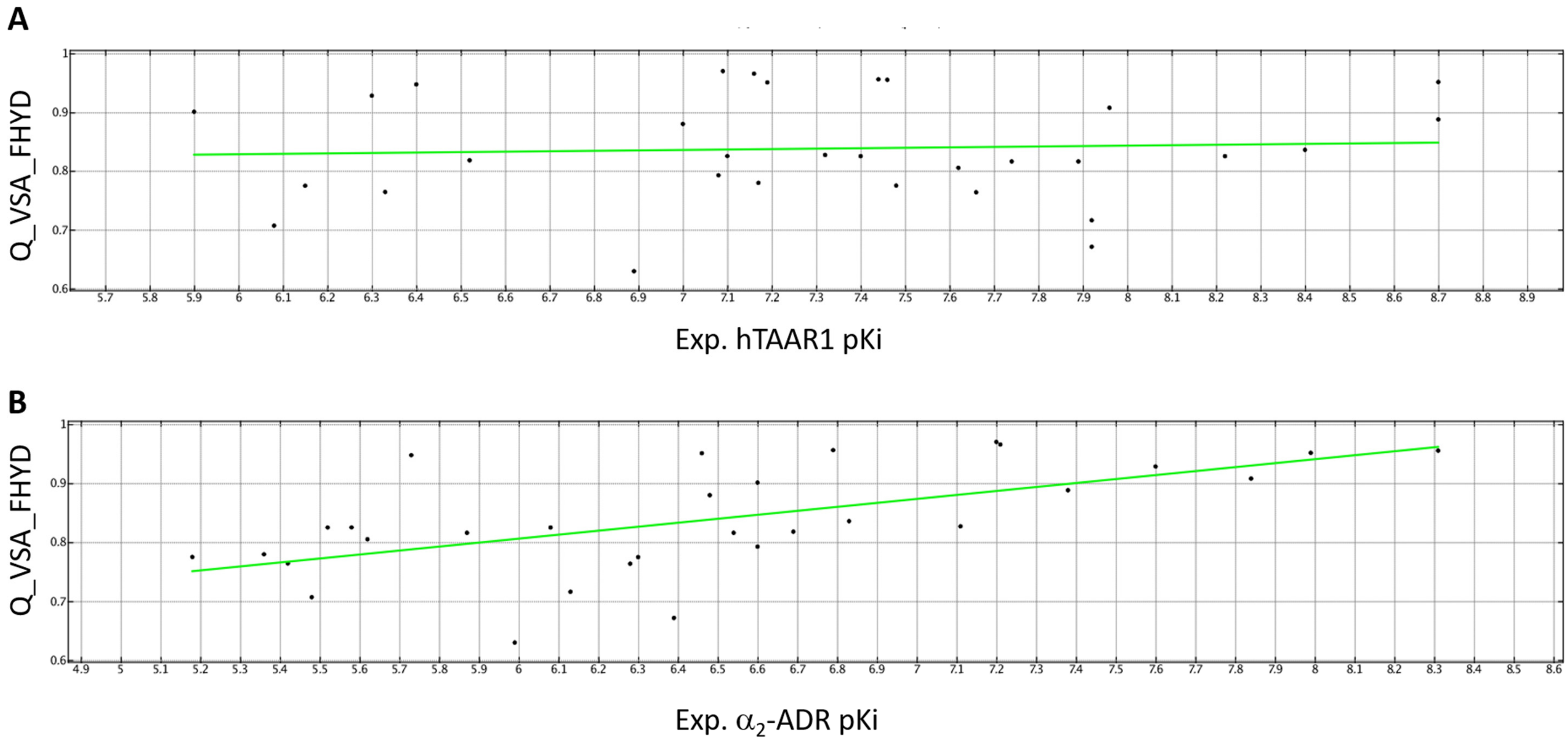

Figure S7. Schematic representation of the opposite role played by the Q_VSA_FHYD descriptor, shared by both models A and B, to influence the compound (*h*TAAR1 and a_2-ADR) binding affinity values. Compounds are represented as dots.

Table S1. Five top scored docking positioning of **5a-5e**, **6a-6e**, **11-54** and the reference agonist **S18616** at the *h*TAAR1 (MOE software). The predicted ΔG value of each protein-ligand complex has been reported, as calculated in terms of final scoring function (S, as Kcal/mol).

Compound	S	E_conf	E_place	E_score1	E_refine	E_score2
**5a**	-5.1695	-18.2208	-15.3929	-7.7987	-11.6232	-5.1695
**5a**	-4.9402	-21.9155	-11.5622	-8.0304	-12.1832	-4.9402
**5a**	-4.7778	-21.0637	-16.6175	-7.5495	-18.4062	-4.7778
**5a**	-4.7573	-18.8261	-20.1722	-9.0459	-5.1190	-4.7573
**5a**	-4.5663	-14.8291	-15.6946	-8.0510	4.8254	-4.5663
**5b**	-5.7383	-14.1695	-19.3227	-7.0323	-4.9471	-5.7383
**5b**	-5.6052	-22.5438	-11.9454	-8.6970	-8.4442	-5.6052
**5b**	-5.5773	-23.7832	-22.1466	-8.3987	-10.3642	-5.5773
**5b**	-5.5106	-29.2996	-20.4277	-7.0394	-2.9662	-5.5106
**5b**	-5.4926	-12.3069	-17.7338	-7.0887	-7.1517	-5.4926
**5c**	-5.6357	-20.9581	-18.7912	-9.5620	-10.2463	-5.6357
**5c**	-5.5748	-25.0866	-14.4636	-9.5479	-11.3133	-5.5748
**5c**	-5.4536	-22.7922	-15.5140	-7.7365	3.8596	-5.4536
**5c**	-5.4508	-26.8145	-15.4513	-9.1423	-3.7877	-5.4508
**5c**	-5.4337	-20.2956	-15.6781	-8.1212	-7.7924	-5.4337
**5d**	-6.2309	-4.6525	-23.8586	-5.9309	-2.8928	-6.2309
**5d**	-6.2308	7.4696	-22.6422	-7.1454	13.1086	-6.2308
**5d**	-6.2161	-2.9400	-15.1797	-6.7809	1.6499	-6.2161
**5d**	-6.1771	-15.4006	-14.3756	-7.1999	8.1803	-6.1771
**5d**	-6.1644	-15.2244	-17.2960	-6.3838	3.0907	-6.1644
**5e**	-7.2043	6.5957	-16.4140	-4.6198	31.5089	-7.2043

**5e**	-5.9711	3.6161	-17.3355	-6.5913	16.9221	-5.9711
**5e**	-5.7338	-3.1095	-20.2515	-3.8649	25.5717	-5.7338
**5e**	-5.6962	15.5878	-27.9443	-6.8042	21.7943	-5.6962
**5e**	-5.6800	10.5990	-12.9053	-4.0603	24.0279	-5.6800
**5f**	-9.0759	84.1545	-20.2790	-6.3740	18.1892	-9.0759
**5f**	-8.6927	83.6578	-15.7717	-4.9193	5.3773	-8.6927
**5f**	-8.6000	86.7519	-13.4784	-5.8091	-0.5324	-8.6000
**5f**	-8.2305	99.6059	-25.1957	-6.6039	21.3119	-8.2305
**5f**	-8.0835	89.5961	-19.5672	-5.9362	11.4194	-8.0835
**6a**	-5.3953	5.7389	-18.6462	-8.3960	-15.9329	-5.3953
**6a**	-4.7282	3.3257	-16.5235	-8.5974	-10.0816	-4.7282
**6a**	-4.4469	2.5288	-13.6917	-8.3754	-16.2506	-4.4469
**6a**	-4.4362	4.3633	-17.8022	-8.3708	-14.5467	-4.4362
**6a**	-4.4188	3.5514	-22.1593	-9.0078	-16.2700	-4.4188
**6b**	-5.4092	3.2340	-22.2534	-7.7432	-5.3643	-5.4092
**6b**	-5.3233	-7.7289	-12.5484	-8.2110	-3.0144	-5.3233
**6b**	-5.3034	8.6078	-14.4162	-7.6342	-7.1388	-5.3034
**6b**	-5.2003	-6.8580	-14.9499	-8.0918	-2.7755	-5.2003
**6b**	-5.1916	-1.1206	-12.7061	-8.3744	-12.3165	-5.1916
**6c**	-5.9041	7.1270	-20.9191	-7.8765	-3.3152	-5.9041
**6c**	-5.5772	1.4818	-16.4811	-8.0756	-9.7957	-5.5772
**6c**	-5.2692	12.5013	-23.5135	-8.6044	-3.2450	-5.2692
**6c**	-5.2339	-0.8363	-12.1743	-7.8086	-3.2521	-5.2339
**6c**	-4.9241	2.2011	-24.1006	-8.8501	-10.9080	-4.9241
**6d**	-6.4847	13.5517	-18.7492	-8.0687	-2.7927	-6.4847
**6d**	-6.3261	17.2291	-12.2230	-7.2478	-6.8761	-6.3261
**6d**	-6.1090	13.6725	-18.2565	-7.0425	-4.6968	-6.1090
**6d**	-5.8187	22.6733	-27.8051	-8.3171	-3.0578	-5.8187
**6d**	-5.7408	17.1991	-21.4535	-5.9925	-5.8574	-5.7408
**6e**	-6.7232	14.0008	-12.2414	-4.7474	16.6205	-6.7232
**6e**	-6.4710	31.6233	-19.7940	-4.9765	9.7882	-6.4710

**6e**	-6.1201	29.3200	-23.5855	-6.0087	22.8018	-6.1201
**6e**	-5.8200	48.9085	-11.2518	-4.2977	19.7191	-5.8200
**6e**	-5.6230	33.2730	-13.4817	-4.6373	19.9673	-5.6230
**6f**	-8.5272	107.1073	-16.2378	-6.7236	-1.5848	-8.5272
**6f**	-8.3479	105.9284	-20.2435	-9.6098	8.5079	-8.3479
**6f**	-8.2120	122.5868	-15.4995	-5.4549	13.4576	-8.2120
**6f**	-8.2082	103.7487	-19.5016	-5.2509	-4.7198	-8.2082
**6f**	-8.0937	118.5481	-16.2313	-5.3252	8.4333	-8.0937
**11**	-5.7090	29.4435	-19.6682	-7.6979	-14.7501	-5.7090
**11**	-5.4144	25.7424	-20.7493	-7.4555	-11.6635	-5.4144
**11**	-5.3315	25.7919	-23.5614	-8.7031	-12.0477	-5.3315
**11**	-5.3078	26.5097	-15.8832	-7.5579	-11.8967	-5.3078
**11**	-5.2500	26.9098	-20.7082	-7.8863	-11.5234	-5.2500
**12**	-6.1454	4.5846	-19.2147	-7.4327	-18.0355	-6.1454
**12**	-5.8119	10.8324	-19.2887	-6.9958	-6.3611	-5.8119
**12**	-5.7567	6.1498	-20.1496	-7.0165	-8.8343	-5.7567
**12**	-5.7313	7.9706	-22.8352	-7.3023	-9.3164	-5.7313
**12**	-5.6702	7.6444	-23.9284	-7.3762	-9.2898	-5.6702
**13**	-5.4040	-7.5283	-21.3915	-7.9391	-11.8903	-5.4040
**13**	-5.2978	-7.7251	-21.1744	-8.0338	-17.0026	-5.2978
**13**	-5.2586	-7.3780	-15.3082	-7.9345	-15.5298	-5.2586
**13**	-4.9231	-9.3209	-15.5753	-7.9255	-15.6561	-4.9231
**13**	-4.8611	-6.8786	-21.1805	-7.4549	-7.8895	-4.8611
**14**	-6.4626	13.3278	-22.1317	-8.7533	-18.7278	-6.4626
**14**	-6.0362	10.5786	-24.7707	-7.9620	-10.7383	-6.0362
**14**	-5.9594	11.7547	-16.0253	-7.4753	-8.2048	-5.9594
**14**	-5.8320	20.3669	-15.2713	-7.5084	2.1454	-5.8320
**14**	-5.7888	15.5874	-17.4586	-7.4924	-4.7848	-5.7888
**15**	-6.7549	1.8752	-23.3285	-7.0698	-15.0008	-6.7549
**15**	-6.6118	-0.4646	-15.6514	-7.3002	-11.8436	-6.6118
**15**	-6.5809	2.5772	-17.2314	-6.4612	10.1483	-6.5809

**15**	-6.2528	0.9143	-19.1425	-6.7599	-12.9160	-6.2528
**15**	-6.2221	-2.2671	-23.5639	-6.5445	-6.2518	-6.2221
**16**	-7.1440	-11.4955	-17.7061	-6.4942	8.9249	-7.1440
**16**	-6.9392	-13.8669	-18.3291	-6.0322	-10.0654	-6.9392
**16**	-6.8404	-19.9071	-16.9745	-6.5779	-5.2914	-6.8404
**16**	-6.6698	-10.4167	-20.2815	-9.0931	0.9210	-6.6698
**16**	-6.3443	-6.7179	-18.7858	-6.6863	9.1221	-6.3443
**17**	-7.3907	52.5896	-16.6319	-3.7547	14.0191	-7.3907
**17**	-7.0762	41.0422	-13.9756	-5.7136	9.3760	-7.0762
**17**	-7.0090	40.6282	-23.6082	-5.9601	37.3648	-7.0090
**17**	-6.8208	51.5054	-20.9760	-4.6030	30.9254	-6.8208
**17**	-6.5617	32.6579	-17.5356	-4.1918	18.9843	-6.5617
**18**	-6.9658	20.8021	-15.6677	-3.5344	5.2737	-6.9658
**18**	-6.6448	20.7317	-16.3956	-4.5367	6.5673	-6.6448
**18**	-6.6294	31.5781	-11.2740	-3.7528	4.3735	-6.6294
**18**	-6.0415	33.6351	-14.6425	-4.9314	4.1108	-6.0415
**18**	-5.7984	31.3644	-18.9887	-4.2499	17.7233	-5.7984
**19**	-6.9677	87.2845	-21.5319	-5.9824	-8.0483	-6.9677
**19**	-6.6508	89.4113	-19.1190	-5.9106	-6.5768	-6.6508
**19**	-6.1856	106.2361	-22.8171	-5.7849	12.1217	-6.1856
**19**	-6.0403	96.5439	-18.3047	-6.6155	6.9887	-6.0403
**19**	-5.9968	91.8334	-22.6399	-7.8948	4.3238	-5.9968
**20**	-7.5258	11.2913	-21.8166	-7.9865	-11.8310	-7.5258
**20**	-7.1799	12.7587	-18.6011	-7.5269	-19.9563	-7.1799
**20**	-7.0693	13.0458	-22.4354	-8.3809	-17.6004	-7.0693
**20**	-6.6850	16.0688	-23.6803	-7.3477	-1.4379	-6.6850
**20**	-6.6562	12.4321	-25.1458	-8.0126	-7.6272	-6.6562
**21**	-6.8669	3.9646	-26.6164	-7.3751	5.1793	-6.8669
**21**	-6.7855	6.0261	-18.1630	-6.7628	-0.8237	-6.7855
**21**	-6.6724	7.6049	-15.6285	-6.6255	7.4249	-6.6724
**21**	-6.3927	2.3062	-23.9178	-6.9732	-1.9235	-6.3927

**21**	-6.2545	5.0963	-19.7187	-6.4777	24.6805	-6.2545
**22**	-6.9699	22.2918	-15.3753	-6.7936	-4.2195	-6.9699
**22**	-6.9692	26.3006	-14.1371	-6.2116	2.2323	-6.9692
**22**	-6.8174	49.5994	-17.2212	-6.4308	14.2583	-6.8174
**22**	-6.6597	33.7985	-18.8914	-5.7997	21.4677	-6.6597
**22**	-6.6221	27.1289	-21.4108	-6.3729	7.9265	-6.6221
**23**	-7.5642	-18.9900	-13.9996	-6.0764	13.4921	-7.5642
**23**	-7.4357	-10.5288	-14.4227	-6.1477	-13.7798	-7.4357
**23**	-7.4089	-25.5283	-18.0524	-6.9771	-6.1266	-7.4089
**23**	-7.2787	-15.9898	-13.9304	-6.0964	25.2386	-7.2787
**23**	-7.0379	-20.3794	-16.0643	-5.8960	23.9315	-7.0379
**24**	-7.3670	-2.4750	-17.3193	-5.4394	-12.8853	-7.3670
**24**	-6.9281	-17.7132	-20.3846	-5.8126	-9.8312	-6.9281
**24**	-6.3662	-14.6660	-17.3525	-5.6328	10.5366	-6.3662
**24**	-6.2868	-11.3076	-19.7575	-5.6213	8.0170	-6.2868
**24**	-6.2675	-16.7269	-20.3474	-6.1909	10.4787	-6.2675
**25**	-7.8486	3.2458	-18.8732	-5.9071	-7.5569	-7.8486
**25**	-7.5222	-15.9165	-22.7457	-6.5862	-0.3337	-7.5222
**25**	-6.8770	-9.6813	-19.5089	-6.9067	4.5640	-6.8770
**25**	-6.8578	25.4552	-17.4251	-5.2570	15.4274	-6.8578
**25**	-6.6790	-10.4764	-19.1989	-6.2277	18.0734	-6.6790
**26**	-6.9376	-9.3366	-18.5366	-5.8124	25.0962	-6.9376
**26**	-6.8056	2.3957	-23.0545	-8.7950	3.2299	-6.8056
**26**	-6.7563	-17.2014	-13.8468	-5.7417	-1.2804	-6.7563
**26**	-6.7378	-23.0396	-19.8893	-6.0405	23.0749	-6.7378
**26**	-6.7248	-21.0151	-13.3140	-5.0265	1.5234	-6.7248
**27**	-7.4888	17.0501	-21.2491	-6.1899	-4.8202	-7.4888
**27**	-7.3580	26.7535	-17.1392	-7.2857	-10.1628	-7.3580
**27**	-7.2921	27.8285	-12.7459	-6.4586	-8.3122	-7.2921
**27**	-6.8127	22.2414	-26.3685	-6.4341	3.8095	-6.8127
**27**	-6.5644	21.5504	-25.4835	-6.7994	-0.2655	-6.5644

**28**	-7.2976	-20.6007	-19.7241	-5.1740	-2.9507	-7.2976
**28**	-7.0139	-3.9735	-18.6085	-5.3661	-2.2493	-7.0139
**28**	-6.9897	-9.0595	-19.6567	-7.3524	0.0144	-6.9897
**28**	-6.7091	-15.1525	-21.1642	-5.7263	9.5314	-6.7091
**28**	-6.3743	-13.9352	-15.0619	-5.4695	6.7566	-6.3743
**29**	-7.1301	-14.0748	-16.4598	-7.5679	9.7341	-7.1301
**29**	-6.7880	-13.4198	-21.1735	-5.9730	-12.3510	-6.7880
**29**	-6.4256	-0.6906	-18.3878	-5.7179	7.6336	-6.4256
**29**	-6.3211	-10.6713	-20.4505	-7.8579	10.9515	-6.3211
**29**	-6.3114	-11.9885	-21.1278	-8.0907	8.9883	-6.3114
**30**	-6.9192	-21.2923	-12.7902	-7.4089	-12.7240	-6.9192
**30**	-6.8965	-19.1422	-10.0821	-5.5674	2.8830	-6.8965
**30**	-6.8485	-17.6830	-8.7993	-4.6989	9.1108	-6.8485
**30**	-6.6031	-7.6384	-16.1648	-6.4406	4.0124	-6.6031
**30**	-6.5161	1.5010	-22.4264	-5.5059	21.0534	-6.5161
**31**	-7.2516	-11.6325	-21.9350	-5.2964	13.7206	-7.2516
**31**	-7.1319	-8.8200	-12.8212	-5.8105	22.5673	-7.1319
**31**	-7.0846	2.2205	-10.7337	-6.1193	9.3931	-7.0846
**31**	-7.0687	-11.1753	-15.0521	-6.7364	20.6687	-7.0687
**31**	-7.0577	-17.5523	-22.4342	-7.2072	15.6324	-7.0577
**32**	-7.9356	27.6877	-17.6383	-4.1913	1.7178	-7.9356
**32**	-7.3214	33.7168	-14.6650	-4.2081	21.1119	-7.3214
**32**	-7.0113	50.0754	-18.5466	-4.2158	52.7006	-7.0113
**32**	-6.7946	30.6555	-22.1453	-4.9065	16.8809	-6.7946
**32**	-6.5793	37.2774	-23.1148	-7.4483	11.9846	-6.5793
**33**	-6.9640	6.9785	-18.3590	-7.0461	-19.1492	-6.9640
**33**	-6.7251	5.9511	-16.8946	-6.9217	-7.6307	-6.7251
**33**	-6.6629	9.9691	-18.4411	-6.9176	-13.6890	-6.6629
**33**	-6.6328	4.5592	-22.2054	-6.8794	-12.6632	-6.6328
**33**	-6.1540	16.1598	-14.7493	-7.0201	1.6893	-6.1540
**34**	-7.3067	2.4457	-18.9596	-6.6431	-18.2095	-7.3067

**34**	-6.8822	-6.0882	-19.1647	-6.7001	-8.4519	-6.8822
**34**	-6.6972	-7.5061	-22.5950	-7.4617	-11.3809	-6.6972
**34**	-6.6733	19.9712	-13.3194	-6.8019	23.3943	-6.6733
**34**	-6.6547	-1.7751	-19.0071	-6.8846	2.1441	-6.6547
**35**	-7.4790	-25.5311	-18.4569	-4.7887	35.3934	-7.4790
**35**	-6.6609	-29.8600	-17.1907	-6.1405	43.5066	-6.6609
**35**	-6.6300	-22.9587	-18.8594	-6.8698	21.6276	-6.6300
**35**	-6.5796	-40.8890	-17.2634	-5.7100	9.4792	-6.5796
**35**	-6.4968	-43.0403	-19.0494	-5.3069	15.4455	-6.4968
**36**	-6.1875	-49.3287	-16.0939	-6.3885	13.7182	-6.1875
**36**	-5.7137	-39.2731	-17.0539	-6.4438	-4.1801	-5.7137
**36**	-5.6017	-47.7943	-14.0281	-6.0964	-0.6161	-5.6017
**36**	-5.5020	-45.5616	-14.5369	-7.0523	13.8918	-5.5020
**36**	-5.4890	-52.1290	-15.1353	-7.0080	6.3151	-5.4890
**37**	-6.5851	-61.0472	-14.5192	-5.5712	10.2112	-6.5851
**37**	-6.4277	-25.4888	-19.7830	-6.5545	18.0433	-6.4277
**37**	-6.4246	-57.8340	-17.6521	-6.8929	1.4875	-6.4246
**37**	-6.4235	-56.8517	-12.2515	-7.5260	-13.4837	-6.4235
**37**	-6.3679	-59.2965	-18.7761	-7.4628	0.5092	-6.3679
**38**	-5.3083	-142.0945	-12.6841	-7.1155	-7.7869	-5.3083
**38**	-5.1815	-135.7909	-9.2237	-5.5435	-6.3058	-5.1815
**38**	-4.8488	-132.6850	-7.5954	-5.9117	8.1609	-4.8488
**38**	-4.8380	-127.0627	-14.1474	-6.0593	13.8880	-4.8380
**38**	-4.8256	-129.0628	-14.7706	-7.5835	9.6282	-4.8256
**39**	-5.3215	-105.9910	-15.6297	-7.6744	0.9608	-5.3215
**39**	-5.2457	-102.3219	-16.7335	-7.4557	-1.4090	-5.2457
**39**	-5.2107	-111.7456	-13.4833	-7.3193	-14.6700	-5.2107
**39**	-5.1875	-110.3807	-16.0767	-6.6105	18.9068	-5.1875
**39**	-5.0764	-105.3330	-12.8936	-7.9981	-6.1866	-5.0764
**49**	-5.2669	16.8708	-19.8830	-8.0641	-12.5071	-5.2669
**49**	-4.9535	20.4225	-16.6438	-7.6979	-14.7143	-4.9535

**49**	-4.9338	22.3600	-17.8494	-7.6705	-9.7148	-4.9338
**49**	-4.9077	20.8152	-16.6727	-7.6482	-13.4018	-4.9077
**49**	-4.7603	17.0036	-19.1916	-7.6223	-12.9514	-4.7603
**50**	-6.3263	36.8180	-23.8001	-7.4244	-1.8658	-6.3263
**50**	-6.2365	35.5761	-22.1739	-7.8108	-8.9523	-6.2365
**50**	-5.7440	43.2290	-11.9291	-7.0000	-9.5796	-5.7440
**50**	-5.3030	39.3780	-18.8912	-8.0380	-9.0851	-5.3030
**50**	-5.1336	36.6016	-19.1499	-8.8704	-8.0785	-5.1336
**51**	-6.4870	23.8828	-15.6876	-6.6502	2.0538	-6.4870
**51**	-6.2588	25.8877	-22.5323	-7.8278	-8.0515	-6.2588
**51**	-6.1905	30.2390	-11.5804	-6.8121	-5.4831	-6.1905
**51**	-5.9857	22.4576	-17.9261	-8.0817	-6.3433	-5.9857
**51**	-5.9833	26.1091	-23.4586	-6.7510	-8.4228	-5.9833
**52**	-6.9199	15.4097	-18.5293	-6.0291	22.3305	-6.9199
**52**	-6.5480	12.4628	-20.7098	-6.0875	17.7096	-6.5480
**52**	-6.3831	13.9484	-20.4280	-7.2490	4.9660	-6.3831
**52**	-6.2719	15.8614	-11.5977	-5.7030	9.3385	-6.2719
**52**	-6.2027	13.9959	-14.5955	-6.3727	1.0225	-6.2027
**53**	-6.8316	11.2652	-16.5366	-6.6175	-0.5447	-6.8316
**53**	-6.7944	21.8153	-17.7039	-7.4494	7.8954	-6.7944
**53**	-6.6836	3.3764	-16.9642	-5.6855	22.9776	-6.6836
**53**	-6.6380	12.2019	-17.2957	-6.1427	3.8562	-6.6380
**53**	-6.6294	7.7140	-18.3127	-5.7353	-2.3184	-6.6294
**54**	-7.4037	22.1949	-13.4854	-5.2514	0.6901	-7.4037
**54**	-6.9224	11.9278	-13.6312	-4.8080	11.5459	-6.9224
**54**	-6.4409	14.9964	-12.5669	-5.4748	2.2407	-6.4409
**54**	-6.2803	16.3959	-11.2060	-4.7610	18.5203	-6.2803
**54**	-6.2716	15.8469	-20.8939	-6.9144	3.7623	-6.2716
**S18616**	-7.2540	-78.9660	-20.1860	-6.6451	-3.8317	-7.2540
**S18616**	-6.5302	-80.7608	-24.7631	-8.4053	1.2911	-6.5302
**S18616**	-6.2843	-75.6964	-23.0394	-9.7113	0.1749	-6.2843

**S18616**	-6.0826	-67.0448	-21.8553	-8.3635	4.8944	-6.0826
**S18616**	-6.0375	-69.6867	-15.9147	-6.5721	6.7722	-6.0375

Table S2. Five top scored docking positioning of **5a-5e**, **6a-6e**, **11-54** and the reference agonist **S18616** at the α_2-ADR (MOE software). The predicted ΔG value of each protein-ligand complex has been reported, as calculated in terms of final scoring function (S, as Kcal/mol).

Compound	S	E_conf	E_place	E_score1	E_refine	E_score2
**5a**	-4.8586	-24.6135	-16.3394	-8.4980	-28.3288	-4.8586
**5a**	-4.6276	-24.5541	-24.3329	-8.5346	-27.1076	-4.6276
**5a**	-4.5228	-24.7498	-14.0069	-8.4813	-24.3698	-4.5228
**5a**	-4.4926	-24.3935	-13.6725	-9.1069	-20.2049	-4.4926
**5a**	-4.3883	-23.1719	-16.4793	-8.4661	-22.3050	-4.3883
**5b**	-5.6235	-35.0779	-18.1036	-9.0295	-25.9453	-5.6235
**5b**	-5.6087	-35.0746	-15.8406	-9.8561	-25.9431	-5.6087
**5b**	-5.5957	-35.0741	-18.3770	-9.4665	-25.9385	-5.5957
**5b**	-5.5213	-34.9964	-20.7360	-9.5987	-29.9274	-5.5213
**5b**	-5.4698	-35.9122	-18.0356	-9.2531	-27.8051	-5.4698
**5c**	-5.6971	-33.9166	-21.4063	-9.4918	-29.9051	-5.6971
**5c**	-5.6018	-34.5350	-17.9618	-10.2308	-30.0033	-5.6018
**5c**	-5.5790	-34.3260	-17.8880	-9.7142	-29.9054	-5.5790
**5c**	-5.1553	-33.7042	-12.4049	-9.3017	-25.1663	-5.1553
**5c**	-5.1022	-34.8817	-19.8985	-9.8771	-26.3130	-5.1022
**5d**	-6.6975	-24.0750	-18.8442	-9.6650	-31.0271	-6.6975
**5d**	-6.6468	-27.0047	-27.9347	-9.4997	-29.5467	-6.6468
**5d**	-6.6046	-25.0672	-24.6577	-9.9896	-30.9893	-6.6046
**5d**	-6.4712	-26.6302	-27.2452	-9.9546	-31.1768	-6.4712
**5d**	-6.4128	-27.8938	-24.6200	-9.7864	-32.4074	-6.4128
**5e**	-7.7755	-27.0518	-30.8449	-9.5500	-30.2690	-7.7755

**5e**	-7.4451	-26.8237	-22.4104	-10.2015	-29.4374	-7.4451
**5e**	-7.2488	-22.0454	-27.3508	-10.1843	-26.4730	-7.2488
**5e**	-7.2126	-18.3991	-24.7323	-10.6521	-32.4382	-7.2126
**5e**	-6.9625	-28.2883	-22.8544	-9.0794	-28.2641	-6.9625
**5f**	-8.5205	67.0418	-22.9174	-9.0811	-19.9307	-8.5205
**5f**	-7.9588	66.6432	-25.5324	-8.7483	-22.6765	-7.9588
**5f**	-7.8949	67.4212	-26.1867	-8.6180	-23.2117	-7.8949
**5f**	-7.7658	69.0728	-21.7586	-9.1882	-21.8958	-7.7658
**5f**	-7.4667	70.6896	-26.0858	-8.6072	-24.0882	-7.4667
**6a**	-4.5923	1.3917	-16.0038	-8.7212	-22.6771	-4.5923
**6a**	-4.5848	1.8910	-13.5773	-9.6259	-18.8813	-4.5848
**6a**	-4.5240	0.6075	-18.4029	-9.0138	-27.3238	-4.5240
**6a**	-4.4712	0.7750	-13.1543	-9.1723	-22.9108	-4.4712
**6a**	-4.4480	1.5839	-19.6700	-9.0532	-28.8079	-4.4480
**6b**	-5.5751	-11.9718	-21.8407	-10.3889	-30.8106	-5.5751
**6b**	-5.3476	-11.0347	-15.4176	-9.2901	-28.0222	-5.3476
**6b**	-5.2181	-11.7669	-15.3975	-9.5230	-24.3644	-5.2181
**6b**	-5.0697	-11.6377	-14.2715	-9.2423	-21.3898	-5.0697
**6b**	-4.9603	-6.4500	-14.4135	-9.4583	-29.6776	-4.9603
**6c**	-5.6721	-8.3077	-21.9437	-9.5122	-31.3923	-5.6721
**6c**	-5.5861	-9.8174	-23.5658	-9.5916	-29.6594	-5.5861
**6c**	-5.4044	-9.0422	-13.5352	-9.1567	-24.4290	-5.4044
**6c**	-5.3778	-9.0981	-15.2839	-9.9294	-24.1277	-5.3778
**6c**	-5.3306	-7.9226	-13.1808	-9.0863	-25.2748	-5.3306
**6d**	-6.6008	-0.3248	-24.8208	-10.3116	-31.2008	-6.6008
**6d**	-6.4652	-0.9956	-20.6028	-9.6940	-29.2753	-6.4652
**6d**	-6.4627	0.9562	-28.1860	-10.4432	-31.7838	-6.4627
**6d**	-6.2677	-0.0595	-12.6899	-9.5491	-30.1310	-6.2677
**6d**	-6.2552	1.6043	-20.9041	-9.4861	-22.5559	-6.2552
**6e**	-7.4403	-3.4952	-19.8243	-8.9487	-27.0887	-7.4403
**6e**	-7.3642	-1.9331	-22.8069	-9.5952	-30.5953	-7.3642

**6e**	-7.1489	7.4743	-27.1922	-9.4821	-30.1815	-7.1489
**6e**	-7.1156	7.6250	-27.2737	-9.3741	-29.4376	-7.1156
**6e**	-7.0922	-4.5141	-26.7742	-9.1401	-30.4486	-7.0922
**6f**	-8.4565	95.5462	-21.9721	-8.8043	-19.7170	-8.4565
**6f**	-8.3076	95.6416	-23.0525	-9.5390	-20.1698	-8.3076
**6f**	-8.2740	99.0312	-22.9908	-9.4941	-21.2341	-8.2740
**6f**	-8.1691	97.1621	-26.3011	-9.4989	-23.8912	-8.1691
**6f**	-7.8912	96.0249	-22.8691	-8.8897	-21.7882	-7.8912
**11**	-5.2893	24.4764	-17.6885	-7.5478	-18.0551	-5.2893
**11**	-4.9647	24.4556	-16.1683	-7.4850	-18.2605	-4.9647
**11**	-4.8230	23.8081	-17.1736	-8.0563	-18.7143	-4.8230
**11**	-4.7963	23.9432	-21.2175	-8.3497	-19.3325	-4.7963
**11**	-4.7555	23.6229	-20.5758	-7.4879	-18.5984	-4.7555
**12**	-5.6061	4.1941	-28.3745	-7.6899	-20.9171	-5.6061
**12**	-5.4827	4.9921	-19.7117	-7.7559	-16.4729	-5.4827
**12**	-5.4537	5.5342	-17.0294	-7.6759	-17.4785	-5.4537
**12**	-5.3983	3.7078	-18.7093	-7.7303	-18.0687	-5.3983
**12**	-5.0632	4.7602	-20.5591	-8.2469	-19.3343	-5.0632
**13**	-5.0472	-10.6894	-17.0682	-7.9067	-19.5178	-5.0472
**13**	-4.6605	-8.5908	-19.0651	-7.8658	-22.1610	-4.6605
**13**	-4.5311	-9.6731	-21.6857	-8.0013	-18.9952	-4.5311
**13**	-4.3904	-10.2218	-19.0629	-9.1749	-21.2946	-4.3904
**13**	-4.3366	-12.0369	-14.5865	-8.5393	-20.6742	-4.3366
**14**	-5.9150	9.0452	-23.4623	-8.6325	-20.7938	-5.9150
**14**	-5.7216	8.3770	-25.7961	-8.0179	-22.1183	-5.7216
**14**	-5.6361	8.7530	-19.5716	-8.0999	-19.6382	-5.6361
**14**	-5.5880	9.4693	-17.8920	-8.6200	-19.8565	-5.5880
**14**	-5.5272	7.5691	-18.4660	-8.1814	-23.6766	-5.5272
**15**	-6.4849	-5.8884	-18.7245	-8.6114	-22.4379	-6.4849
**15**	-6.1890	-3.7572	-22.5364	-8.1282	-20.4216	-6.1890
**15**	-6.0194	-5.8803	-21.2829	-8.2021	-21.6515	-6.0194

**15**	-6.0008	-5.2049	-24.9710	-8.0945	-24.7719	-6.0008
**15**	-5.9919	-5.1193	-24.8000	-8.0240	-22.8502	-5.9919
**16**	-6.4794	-24.6931	-23.1690	-8.6052	-19.6042	-6.4794
**16**	-6.3793	-22.3213	-21.0684	-8.3938	-22.5907	-6.3793
**16**	-6.3629	-22.8980	-17.0807	-9.0425	-24.2801	-6.3629
**16**	-6.2030	-27.9002	-19.7858	-9.0118	-26.2401	-6.2030
**16**	-6.1590	-26.6745	-20.6292	-8.6154	-18.8615	-6.1590
**17**	-8.2375	15.5268	-20.8035	-8.6664	-25.6318	-8.2375
**17**	-8.0483	15.6743	-21.9344	-8.1806	-27.1242	-8.0483
**17**	-7.9764	13.1765	-24.4243	-9.4227	-25.8361	-7.9764
**17**	-7.9255	12.7603	-26.3356	-8.6168	-28.9424	-7.9255
**17**	-7.8681	18.8517	-21.1059	-8.6939	-17.9974	-7.8681
**18**	-8.1808	11.4209	-16.4293	-9.2084	-23.9614	-8.1808
**18**	-8.0580	13.0149	-26.8273	-8.3839	-23.4903	-8.0580
**18**	-7.8717	11.2027	-22.1308	-8.4057	-24.3585	-7.8717
**18**	-7.6858	10.0537	-19.3899	-9.0605	-26.8126	-7.6858
**18**	-7.5633	11.2682	-19.7094	-9.1780	-23.6707	-7.5633
**19**	-6.4273	86.0574	-22.2260	-8.7334	-24.3412	-6.4273
**19**	-6.4140	81.3789	-22.6699	-8.8874	-24.7326	-6.4140
**19**	-6.3230	82.9245	-20.4913	-8.7655	-21.3012	-6.3230
**19**	-6.1992	84.2436	-22.3513	-8.4652	-24.2098	-6.1992
**19**	-6.1686	82.8313	-23.7100	-8.9442	-24.4580	-6.1686
**20**	-6.3690	7.6975	-22.5205	-8.5778	-26.4197	-6.3690
**20**	-6.3401	8.6907	-27.4004	-8.2581	-23.8456	-6.3401
**20**	-5.8429	8.8081	-23.5738	-8.2467	-24.8103	-5.8429
**20**	-5.6251	8.6018	-22.7828	-8.8563	-24.1714	-5.6251
**20**	-5.6100	8.5109	-25.3998	-8.2508	-16.6768	-5.6100
**21**	-6.8500	-2.7011	-23.7407	-9.8279	-23.5773	-6.8500
**21**	-6.7064	-3.6583	-22.0166	-9.3438	-23.1503	-6.7064
**21**	-6.5792	-3.0447	-25.2036	-8.4807	-24.6228	-6.5792
**21**	-6.4927	-0.6995	-19.1569	-9.0490	-22.0617	-6.4927

**21**	-6.4824	-3.7909	-26.1106	-9.2498	-26.1341	-6.4824
**22**	-6.7659	6.6827	-25.1337	-8.7522	-25.7750	-6.7659
**22**	-6.6615	2.3951	-25.2083	-9.1748	-28.3974	-6.6615
**22**	-6.5301	8.6240	-31.5079	-8.6680	-24.8482	-6.5301
**22**	-6.5279	10.8462	-25.0707	-8.8380	-25.2320	-6.5279
**22**	-6.5259	9.5229	-26.0648	-8.9508	-25.9095	-6.5259
**23**	-7.0465	-29.0430	-19.5709	-8.4389	-21.4439	-7.0465
**23**	-6.7754	-29.8078	-30.5248	-8.4764	-28.5234	-6.7754
**23**	-6.7675	-31.4773	-20.4636	-9.1517	-20.3325	-6.7675
**23**	-6.7038	-28.9750	-25.6602	-9.6049	-20.5462	-6.7038
**23**	-6.6621	-34.2820	-18.4518	-8.5893	-28.0422	-6.6621
**24**	-6.9405	-21.8172	-20.0137	-8.6395	-26.5432	-6.9405
**24**	-6.9078	-28.6335	-23.6255	-8.7572	-20.9000	-6.9078
**24**	-6.8661	-30.0145	-21.5256	-8.5202	-21.4679	-6.8661
**24**	-6.6388	-27.7759	-26.5501	-9.5617	-27.7064	-6.6388
**24**	-6.6353	-26.7619	-23.1236	-9.1385	-23.8164	-6.6353
**25**	-7.7120	-16.5462	-22.6576	-8.7023	-9.1316	-7.7120
**25**	-7.6023	-23.3050	-24.0619	-8.9729	-25.6927	-7.6023
**25**	-7.1223	-24.3009	-26.0583	-8.7093	-22.7181	-7.1223
**25**	-7.1162	-26.0173	-19.4638	-8.6300	-26.4235	-7.1162
**25**	-7.1073	-23.4272	-17.1189	-8.8531	-19.6431	-7.1073
**26**	-7.3091	-48.4599	-20.2525	-8.9794	-25.3975	-7.3091
**26**	-7.2557	-43.8436	-25.0952	-9.3903	-20.9577	-7.2557
**26**	-7.1310	-47.5892	-26.3670	-9.5929	-22.0982	-7.1310
**26**	-7.0794	-47.9655	-20.9768	-8.7592	-21.0231	-7.0794
**26**	-7.0100	-41.7263	-23.2032	-9.2199	-22.1466	-7.0100
**27**	-6.9431	9.6766	-27.7418	-8.4787	-24.9387	-6.9431
**27**	-6.6521	14.4414	-25.5952	-8.4380	-16.4351	-6.6521
**27**	-6.6221	9.3678	-19.5618	-8.3047	-21.5235	-6.6221
**27**	-6.5012	8.9346	-19.9677	-8.4928	-27.3237	-6.5012
**27**	-6.4085	8.4955	-27.0645	-9.0949	-23.0589	-6.4085

**28**	-7.1420	-28.3034	-21.3577	-8.8130	-22.8444	-7.1420
**28**	-7.0870	-28.1973	-25.7588	-8.7902	-25.0603	-7.0870
**28**	-6.9778	-31.1672	-20.3190	-8.9944	-20.5990	-6.9778
**28**	-6.9118	-25.6494	-19.1445	-8.7814	-21.3153	-6.9118
**28**	-6.8751	-29.0449	-25.1623	-8.8561	-21.7327	-6.8751
**29**	-7.2080	-25.2156	-20.0452	-9.0478	-19.9407	-7.2080
**29**	-7.1689	-23.6104	-26.1553	-9.3239	-23.1209	-7.1689
**29**	-7.0833	-25.2451	-17.7026	-9.4114	-26.3755	-7.0833
**29**	-6.9566	-22.4460	-20.4634	-9.1144	-23.7898	-6.9566
**29**	-6.9469	-19.8292	-25.8112	-8.9191	-24.9063	-6.9469
**30**	-7.3630	-33.1237	-23.5025	-9.2702	-18.5080	-7.3630
**30**	-7.0617	-29.6795	-27.7426	-9.9727	-27.2527	-7.0617
**30**	-6.5697	-31.1752	-21.2295	-8.8782	-19.1557	-6.5697
**30**	-6.4474	-33.9640	-26.6247	-10.7990	-30.0843	-6.4474
**30**	-6.3631	-25.4738	-24.4466	-9.0089	-9.9808	-6.3631
**31**	-7.0555	-32.6103	-26.3370	-8.4073	-27.0119	-7.0555
**31**	-6.6533	-29.2583	-20.1706	-8.4779	-24.6192	-6.6533
**31**	-6.6374	-30.1124	-31.8876	-9.4331	-26.1290	-6.6374
**31**	-6.6122	-34.4360	-23.2535	-8.5433	-28.9754	-6.6122
**31**	-6.4535	-31.0395	-23.5407	-8.4279	-26.0056	-6.4535
**32**	-7.9596	10.6751	-27.8576	-8.5502	-32.4183	-7.9596
**32**	-7.4148	14.4770	-21.7666	-8.8117	-27.2807	-7.4148
**32**	-7.2023	11.0701	-20.3251	-9.0098	-30.1122	-7.2023
**32**	-7.1874	11.3289	-22.5557	-9.3997	-34.9901	-7.1874
**32**	-7.1791	11.3595	-15.0901	-8.7211	-35.0485	-7.1791
**33**	-6.3158	1.1002	-21.4893	-8.4109	-22.1087	-6.3158
**33**	-6.2099	2.8879	-21.1667	-8.4180	-22.5104	-6.2099
**33**	-5.9487	4.1585	-20.6245	-8.5666	-24.8364	-5.9487
**33**	-5.8188	2.4838	-24.3193	-8.5676	-22.5270	-5.8188
**33**	-5.7877	2.9869	-18.0356	-8.5083	-20.5043	-5.7877
**34**	-6.8627	-11.9049	-20.8585	-8.5721	-25.5698	-6.8627

**34**	-6.3844	-8.5546	-18.5868	-8.6418	-23.9540	-6.3844
**34**	-6.3648	-10.3306	-17.4247	-8.8636	-24.3822	-6.3648
**34**	-6.2867	-11.0908	-28.6052	-8.6405	-20.5439	-6.2867
**34**	-6.2282	-8.2014	-23.6725	-8.9825	-21.5663	-6.2282
**35**	-7.2505	-61.8967	-22.8761	-8.4210	-27.3701	-7.2505
**35**	-7.0719	-63.5825	-24.5839	-8.8749	-23.2964	-7.0719
**35**	-7.0673	-60.0247	-27.3402	-8.7845	-25.1516	-7.0673
**35**	-7.0603	-62.0371	-21.2994	-8.8864	-24.9197	-7.0603
**35**	-6.9746	-60.7380	-27.7190	-9.7019	-25.8681	-6.9746
**36**	-5.5456	-58.1209	-22.9800	-8.6841	-24.0728	-5.5456
**36**	-5.3522	-57.0890	-28.8481	-8.4498	-21.7855	-5.3522
**36**	-5.3061	-58.7202	-17.9359	-8.3096	-24.3760	-5.3061
**36**	-5.2991	-56.3874	-18.8060	-8.4978	-21.5264	-5.2991
**36**	-5.2873	-59.4140	-21.6832	-8.4145	-25.4309	-5.2873
**37**	-6.0881	-67.7509	-24.4429	-9.2699	-22.0982	-6.0881
**37**	-5.9731	-73.9127	-19.0932	-8.6246	-29.7191	-5.9731
**37**	-5.9525	-71.2678	-21.8601	-8.6319	-29.0978	-5.9525
**37**	-5.9404	-72.1029	-24.7118	-8.3766	-27.7368	-5.9404
**37**	-5.8757	-73.7415	-17.6358	-8.5947	-25.6477	-5.8757
**38**	-5.5352	-145.3073	-17.0633	-8.6844	-19.8623	-5.5352
**38**	-4.9667	-140.8827	-19.8235	-8.6551	-28.0095	-4.9667
**38**	-4.9584	-143.9345	-19.4633	-9.6520	-17.9319	-4.9584
**38**	-4.8859	-141.0978	-20.1711	-8.7603	-25.9320	-4.8859
**38**	-4.8726	-141.7802	-21.7738	-8.5544	-26.7487	-4.8726
**39**	-5.3322	-118.9785	-9.1725	-9.3137	-18.6062	-5.3322
**39**	-4.8993	-117.8070	-11.7296	-8.5104	-21.8773	-4.8993
**39**	-4.8931	-119.4353	-17.4423	-8.3612	-29.8660	-4.8931
**39**	-4.7796	-118.8688	-17.9774	-8.7614	-25.3359	-4.7796
**39**	-4.6111	-119.4369	-16.3157	-8.7313	-30.7797	-4.6111
**49**	-4.3046	17.5077	-15.5436	-7.7167	-19.8817	-4.3046
**49**	-4.2884	17.4115	-14.8656	-8.1710	-19.0041	-4.2884

**49**	-4.2882	14.4340	-19.1156	-8.2046	-21.4504	-4.2882
**49**	-4.1990	18.8178	-18.3678	-8.2389	-18.3937	-4.1990
**49**	-4.1912	20.5396	-15.3888	-8.1015	-19.5023	-4.1912
**50**	-5.6176	36.2641	-15.1168	-7.9854	-20.0901	-5.6176
**50**	-5.6173	37.6235	-19.1195	-7.8075	-21.5770	-5.6173
**50**	-5.4932	37.4128	-20.6806	-7.8797	-21.2514	-5.4932
**50**	-5.2406	35.5924	-15.8339	-7.9518	-20.3255	-5.2406
**50**	-5.2179	37.1503	-16.7410	-7.7157	-22.4346	-5.2179
**51**	-6.0491	19.7803	-19.0691	-8.1402	-23.1358	-6.0491
**51**	-6.0439	20.2981	-22.7599	-8.2127	-23.2525	-6.0439
**51**	-6.0204	21.9774	-20.6010	-8.8690	-21.8055	-6.0204
**51**	-5.8278	23.8223	-24.2422	-8.2092	-23.0137	-5.8278
**51**	-5.7679	23.6413	-15.0923	-8.5685	-21.6630	-5.7679
**52**	-6.3798	3.9218	-14.0076	-8.4794	-21.3763	-6.3798
**52**	-6.3741	-3.1675	-20.8488	-8.7941	-22.1811	-6.3741
**52**	-6.1751	-3.0134	-22.5094	-8.6615	-21.4697	-6.1751
**52**	-6.1436	2.3686	-18.8041	-8.8918	-23.5124	-6.1436
**52**	-6.0845	6.1998	-23.4263	-9.2607	-20.8541	-6.0845
**53**	-6.6388	-0.1960	-14.7378	-9.0941	-15.9828	-6.6388
**53**	-6.5780	-7.8056	-20.5304	-9.3202	-23.6089	-6.5780
**53**	-6.5190	-0.1792	-23.3101	-8.4270	-16.3114	-6.5190
**53**	-6.5175	-8.3566	-21.5743	-8.5800	-24.3277	-6.5175
**53**	-6.5070	-7.5486	-18.3678	-8.8382	-24.5050	-6.5070
**54**	-7.1372	-2.0929	-22.1443	-8.7294	-23.8655	-7.1372
**54**	-6.7926	4.8865	-15.2122	-9.0549	-21.4526	-6.7926
**54**	-6.5545	3.3080	-23.7167	-8.8303	-25.0563	-6.5545
**54**	-6.4753	-3.5967	-21.5505	-8.9400	-22.3819	-6.4753
**54**	-6.4619	-2.7573	-17.3143	-9.7629	-21.0312	-6.4619
**S18616**	-6.7109	-85.1541	-26.2111	-8.7237	-25.7039	-6.7109
**S18616**	-6.5137	-81.8289	-22.3768	-8.6693	-24.3697	-6.5137
**S18616**	-6.4083	-84.2816	-21.0786	-8.6710	-23.3563	-6.4083

**S18616**	-6.3762	-81.4119	-19.1685	-9.1557	-23.2755	-6.3762
**S18616**	-6.3178	-76.6740	-22.7904	-9.8164	-15.2940	-6.3178

Table S3. Chemical structure of the dual acting *h*TAAR1 and α_2ADR ligands. The corresponding binding affinity values are also reported.

Compound	TAAR1 Ki (nM)	α_2-ADR /hTAAR1 Ki	α_2-ADR Ki (nM)	Chemical Structure
**5a**	1640.00	0.06	98.40	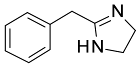
**5b**	500.00	0.05	25.00	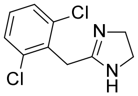
**5c**	82.00	0.77	63.14	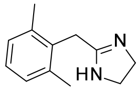
**5d**	300.00	0.68	204.00	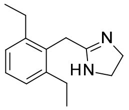
**5e**	825.00	4.00	3300.00	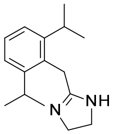
**6a**	400.00	4.70	1880.00	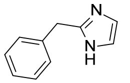
**6b**	1390.00	0.70	973.00	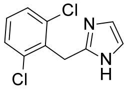
**6c**	36.00	4.50	162.00	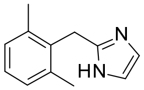

**6d**	24.00	100.00	2400.00	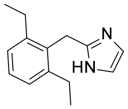
**6e**	630.00	14.00	8820.00	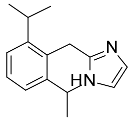
**11**	20.00	2.50	50.00	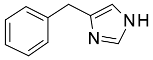
**12**	2.00	5.10	10.20	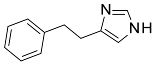
**13**	65.00	5.30	344.50	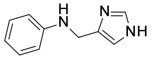
**14**	11.00	1.60	17.60	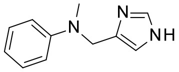
**15**	48.00	1.60	76.80	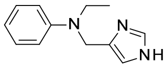
**16**	22.00	23.70	521.40	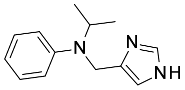
**17**	32.00	4.00	128.00	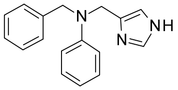
**18**	69.00	0.90	62.10	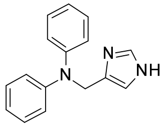
**19**	100.00	3.30	330.00	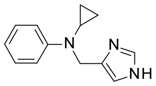
**20**	35.00	0.14	4.90	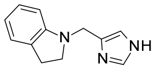

**21**	12.00	4.00	48.00	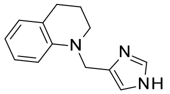
**22**	40.00	76.00	3040.00	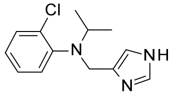
**23**	4.00	72.00	288.00	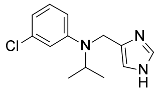
**24**	6.00	140.00	840.00	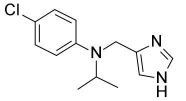
**25**	4.00	71.00	284.00	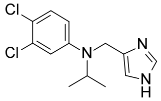
**26**	4.00	37.00	148.00	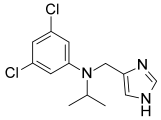
**27**	18.00	16.00	288.00	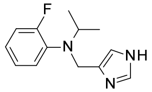
**28**	12.00	34.00	408.00	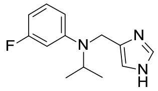
**29**	13.00	104.00	1352.00	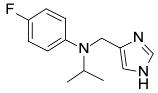
**30**	5.00	67.00	335.00	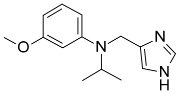

**31**	80.00	33.00	2640.00	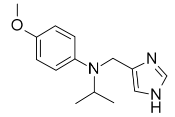
**32**	128.00	8.00	1024.00	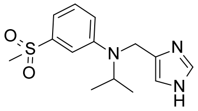
**33**	11.00	1.30	14.30	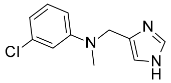
**34**	2.00	21.00	42.00	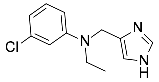
**35**	33.00	200.00	6600.00	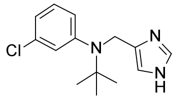
**36**	195.00	12.00	2340.00	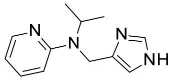
**37**	12.00	62.00	744.00	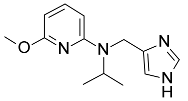
**38**	100.00	33.00	3300.00	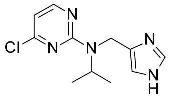
**39**	84.00	3.00	252.00	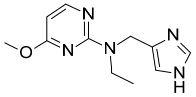
**49**	4250.00	0.20	850.00	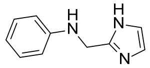
**50**	1270.00	0.20	254.00	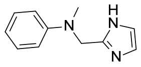

**51**	710.00	0.70	497.00	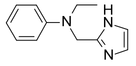
**52**	470.00	8.00	3760.00	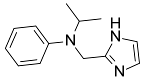
**53**	68.00	64.00	4352.00	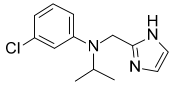
**54**	138.00	22.00	3036.00	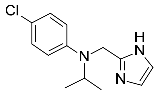

Table S4. The predicted (Pred. pKi) and experimental (Exp. pKi) *h*TAAR1 binding affinity values of the compounds (Comp.) herein explored are reported, in tandem with the collected descriptors. The compounds included in the test set are underlined along the first column.

Comp.	Exp. hTAAR1 pKi	GCUT_SMR_0	E	dipoleY	dipoleZ	DCASA	Q_RPC-	Q_VSA_FHYD	SlogP_VSA4	vsurf_EDmin1	vsurf_IW5	Pred. hTAAR1 pKi	Residual
**5a**	5.79	-0.5048	31.5871	-0.2109	0.1291	157.4579	0.1929	0.9660	3.1856	-2.5019	2.5918	5.81	0.02
**5b**	6.30	-0.5048	35.6559	-0.2837	0.0583	105.3684	0.2856	0.9300	3.1856	-2.8846	4.3883	6.43	0.13
**5c**	7.09	-0.5049	36.8534	-0.1856	0.1142	197.4412	0.1676	0.9717	9.5567	-2.4239	3.2398	6.65	-0.44
**5d**	6.52	-0.5164	40.9543	-0.1532	0.1450	309.1655	0.1460	0.8196	9.5567	-2.2841	3.4189	6.58	0.06
**5e**	6.08	-0.5222	51.2710	-0.1713	0.1310	420.1130	0.1252	0.7085	9.5567	-2.3425	3.5809	6.28	0.20
**6a**	6.40	-0.4721	17.4561	-0.3373	0.1267	39.2795	0.1286	0.9492	5.5135	-2.3605	3.1705	6.70	0.30
**6b**	5.86	-0.4719	22.6175	-0.4081	0.0531	7.5834	0.1471	0.9579	5.5135	-2.9112	4.4446	7.19	1.33
**6c**	7.44	-0.4847	22.7412	-0.2384	0.0291	77.0173	0.1077	0.9578	11.8846	-2.3530	3.5965	7.60	0.16
**6d**	7.62	-0.5164	27.2421	-0.2481	0.1213	187.1044	0.1024	0.8067	11.8846	-2.2929	3.8201	7.56	-0.06
**6e**	6.20	-0.5222	37.6495	-0.2553	0.0813	279.6188	0.0881	0.6968	11.8846	-2.3479	3.6272	7.19	0.99
**11**	7.70	-0.4682	19.6551	0.2232	0.3121	39.5252	0.1436	0.9484	5.9423	-2.7064	3.6205	8.35	0.65
**12**	8.70	-0.5062	19.8977	0.2076	0.3165	107.3042	0.1291	0.9532	5.9423	-2.8273	3.5625	8.22	-0.48
**13**	7.19	-0.4661	27.3867	0.2779	0.3305	118.9644	0.1727	0.9526	2.7567	-2.4910	3.1167	7.28	0.09
**14**	7.96	-0.4777	35.6927	0.2773	0.4286	129.5135	0.1664	0.9021	2.7567	-2.4044	4.3910	7.55	-0.41

**15**	7.32	-0.5075	34.0067	0.2784	0.4457	172.0687	0.1479	0.8286	2.7567	-2.4756	4.3415	7.70	0.38
**16**	7.66	-0.5159	36.9333	0.2558	0.3741	227.1855	0.1336	0.7652	2.7567	-2.4805	4.5911	7.58	-0.08
**17**	7.49	-0.4747	55.3304	0.2831	0.3820	172.2133	0.1204	0.9263	5.9423	-2.4080	5.0069	7.55	0.06
**18**	7.16	-0.4640	59.3388	0.3181	0.1707	121.0741	0.1098	0.9673	2.7567	-2.5218	4.9119	7.04	-0.12
**19**	7.00	-0.4690	38.1040	0.2488	0.4033	211.4028	0.1293	0.8814	2.7567	-2.6171	4.1916	7.34	0.34
**20**	7.46	-0.5048	37.7179	0.2324	0.3856	175.0137	0.1665	0.9569	5.9423	-2.4391	4.6835	7.73	0.27
**21**	7.92	-0.5144	35.4751	0.2615	0.4461	224.3391	0.1507	0.9603	5.9423	-2.6025	4.6638	7.88	-0.04
**22**	7.40	-0.5158	44.5035	0.1838	0.4414	57.1006	0.1647	0.8266	2.7567	-2.8280	4.0049	7.70	0.30
**23**	8.40	-0.5159	36.3419	0.1463	0.6620	32.7312	0.1450	0.8266	2.7567	-2.9375	4.0920	8.19	-0.21
**24**	8.22	-0.5159	37.2205	0.2702	0.5672	23.7212	0.1452	0.8266	2.7567	-2.8690	4.4702	8.44	0.22
**25**	8.40	-0.5158	39.0151	0.2055	0.8128	145.5844	0.1511	0.8373	2.7567	-3.1213	4.2703	8.21	-0.19
**26**	8.40	-0.5158	35.5056	0.2912	0.5749	144.4861	0.1490	0.8373	2.7567	-3.0699	4.5032	8.32	-0.08
**27**	7.74	-0.5159	40.8565	0.0533	0.4840	160.3320	0.1457	0.8176	2.7567	-2.5605	4.2162	7.19	-0.55
**28**	7.92	-0.5159	33.3279	0.1153	0.7269	159.9934	0.1355	0.6726	2.7567	-2.7422	4.0707	8.04	0.12
**29**	7.89	-0.5159	36.8043	0.2724	0.6086	155.0814	0.1441	0.8176	2.7567	-2.6938	4.3367	7.97	0.08
**30**	8.30	-0.5159	44.3150	0.3425	0.4258	281.6604	0.1247	0.6961	2.7567	-2.8822	3.7816	7.63	-0.67
**31**	7.10	-0.5159	48.0157	0.3007	0.2334	281.4287	0.1342	0.8267	2.7567	-2.8415	3.8066	7.04	-0.06
**32**	6.89	-0.5159	18.7812	-0.2198	0.7839	359.2455	0.1709	0.6311	2.7567	-2.6959	3.2562	6.97	0.08
**33**	7.96	-0.4776	35.0484	0.1958	0.6975	28.5787	0.1689	0.9096	2.7567	-2.8021	4.0481	8.06	0.10
**34**	8.70	-0.5074	36.6627	0.1293	0.5517	22.5185	0.1566	0.8898	2.7567	-2.5780	4.8064	7.93	-0.77
**35**	7.48	-0.5191	46.8478	0.0650	0.4660	23.7223	0.1375	0.7766	2.7567	-2.7308	4.8160	7.81	0.33
**36**	6.71	-0.5158	34.0791	0.2448	0.1901	212.0978	0.1190	0.6909	2.7567	-2.6528	3.2740	7.39	0.68
**37**	7.92	-0.5158	37.2112	0.3324	0.2404	268.9462	0.1117	0.7176	2.7567	-2.6913	4.0007	7.58	-0.34
**38**	7.00	-0.5157	29.1544	0.2132	0.3551	462.3309	0.1342	0.7344	2.7567	-2.9915	2.1633	6.69	-0.31
**39**	7.08	-0.5075	32.5707	0.4336	0.2463	331.3762	0.1314	0.7941	2.7567	-2.7324	2.4740	7.13	0.05
**49**	5.37	-0.4695	27.2743	-0.2927	0.1517	93.9851	0.1716	0.9073	2.3279	-2.4231	3.3491	6.10	0.73
**50**	5.90	-0.4781	35.1841	-0.3034	0.2338	104.3671	0.1657	0.9028	2.3279	-2.3549	3.8473	6.04	0.14
**51**	6.15	-0.5075	33.3358	-0.3183	0.2821	145.9252	0.1519	0.7763	2.3279	-2.3956	3.8095	6.26	0.11
**52**	6.33	-0.5159	35.9727	-0.3244	0.2784	177.5581	0.1383	0.7657	2.3279	-2.3224	3.4208	5.96	-0.37
**53**	7.17	-0.5159	35.7515	-0.0575	0.2377	14.3486	0.1402	0.7812	2.3279	-2.2991	4.0105	7.06	-0.11
**54**	6.86	-0.5159	36.6405	-0.2461	0.4066	8.5404	0.1409	0.8271	2.3279	-2.4028	3.9164	6.77	-0.09

Table S5. The predicted (Pred. pKi) and experimental (Exp. pKi) α_2-ADR binding affinity values of the compounds (Comp.) herein explored are reported, in tandem with the collected descriptors. The compounds included in the test set are underlined along the first column.

Comp.	Exp. α2-ADR pKi	GCUT_SMR_1	balabanJ	E_tor	Q_VSA_FHYD	Q_VSA_PNEG	Q_VSA_POL	vsa_other	SlogP_VSA3	vsurf_ID1	vsurf_ID7	vsurf_IW4	Pred. α2-ADR Ki	Residual
**5a**	7.01	-0.1920	1.7133	6.3513	0.9660	5.8195	5.8195	11.1906	36.8792	0.4434	0.6388	2.3317	7.05	7.01
**5b**	7.60	-0.1522	1.8792	6.2372	0.9300	5.8195	14.4385	11.1906	36.8792	0.3928	0.9248	3.3062	7.97	7.60
**5c**	7.20	-0.1506	1.8792	7.9738	0.9717	5.8195	5.8195	11.1906	36.8792	0.4681	0.7490	2.9026	7.07	7.20
**5d**	6.69	-0.1502	1.9471	7.9827	0.8196	43.3148	43.3148	11.1906	36.8792	0.4608	0.8987	2.9886	6.41	6.69
**5e**	5.48	-0.1487	2.0545	8.5903	0.7085	80.8102	80.8102	11.1906	36.8792	0.4280	0.9322	3.1916	5.58	5.48
**6a**	5.73	-0.2094	1.7159	3.9091	0.9492	0.0000	8.6190	11.1906	0.0000	0.4257	0.4994	2.6134	6.50	5.73
**6b**	6.01	-0.1759	1.8811	3.6180	0.9579	0.0000	8.6190	11.1906	0.0000	0.3239	0.6892	3.4183	6.69	6.01
**6c**	6.79	-0.1726	1.8811	5.2639	0.9578	0.0000	8.6190	11.1906	0.0000	0.4211	0.3080	3.1153	6.18	6.79
**6d**	5.62	-0.1717	1.9484	5.2933	0.8067	37.4954	46.1144	11.1906	0.0000	0.4083	0.5363	3.1678	5.60	5.62
**6e**	5.05	-0.1691	2.0555	5.8089	0.6968	74.9907	83.6097	11.1906	0.0000	0.3610	0.4533	3.3044	4.63	5.05
**11**	7.30	-0.2161	1.7164	3.3058	0.9484	0.1369	8.7559	24.7905	0.0000	0.5883	0.6782	3.4603	7.29	7.30
**12**	7.99	-0.2161	1.6145	0.0625	0.9532	0.1369	8.7559	24.7905	0.0000	0.5427	0.8767	3.3721	7.70	7.99
**13**	6.46	-0.2173	1.6145	3.6814	0.9526	0.2738	8.8928	24.7905	0.0000	0.6181	0.1463	2.5496	6.61	6.46
**14**	7.75	-0.1975	1.7368	4.1318	0.9021	12.3918	21.0108	24.7905	0.0000	0.6273	0.4429	4.0897	6.96	7.75
**15**	7.11	-0.1963	1.8018	4.0616	0.8286	31.1395	39.7585	24.7905	18.4396	0.5725	0.4914	4.1262	7.15	7.11
**16**	6.28	-0.1938	1.8802	4.9837	0.7652	49.8872	58.5062	24.7905	0.0000	0.6438	0.5367	4.3255	6.16	6.28
**17**	6.89	-0.2161	1.5574	5.3588	0.9263	12.3918	21.0108	24.7905	0.0000	0.5629	0.4175	4.8839	7.36	6.89
**18**	7.21	-0.2161	1.6255	8.8673	0.9673	0.1369	8.7559	24.7905	0.0000	0.5888	0.4177	4.6235	7.33	7.21
**19**	6.48	-0.2161	1.5695	12.1702	0.8814	17.5395	26.1585	24.7905	0.0000	0.5249	0.5755	4.0537	6.96	6.48
**20**	8.31	-0.1857	1.5747	7.4637	0.9569	0.1369	8.7559	24.7905	18.4396	0.5831	0.3270	4.4199	7.92	8.31
**21**	7.32	-0.1858	1.5975	4.4908	0.9603	0.1369	8.7559	24.7905	18.4396	0.5441	0.4096	4.4360	8.05	7.32
**22**	5.52	-0.1802	1.9531	15.3712	0.8266	37.6322	46.2513	24.7905	0.0000	0.4334	0.5836	3.9053	5.85	5.52
**23**	6.54	-0.1748	1.9124	7.3751	0.8266	37.6322	46.2513	24.7905	0.0000	0.4706	0.6459	3.8533	6.21	6.54
**24**	6.08	-0.1797	1.8776	7.4955	0.8266	37.6322	46.2513	24.7905	0.0000	0.4711	0.6446	4.2023	6.36	6.08
**25**	6.55	-0.1647	1.9273	7.4668	0.8373	37.6322	46.2513	24.7905	0.0000	0.4115	0.6399	4.0779	6.23	6.55
**26**	6.83	-0.1460	1.9562	7.3479	0.8373	37.6322	46.2513	24.7905	0.0000	0.4088	0.5248	4.2847	6.14	6.83

**27**	6.54	-0.1937	1.9531	9.6877	0.8176	37.6322	46.2513	24.7905	0.0000	0.8199	1.2406	3.5093	6.70	6.54
**28**	6.39	-0.1935	1.9124	9.2588	0.6726	74.3970	83.0160	24.7905	0.0000	0.9619	1.2894	3.4352	6.13	6.39
**29**	5.87	-0.1933	1.8776	8.9709	0.8176	37.6322	46.2513	24.7905	0.0000	0.7650	0.9268	2.9975	6.34	5.87
**30**	6.47	-0.1906	1.9154	9.2557	0.6961	66.9007	85.5197	24.7905	0.0000	0.5243	0.9115	3.0937	6.31	6.47
**31**	5.58	-0.1968	1.8571	7.2616	0.8267	40.1360	48.7550	24.7905	0.0000	0.2485	0.3474	2.5761	5.48	5.58
**32**	5.99	-0.1746	1.9757	46.6133	0.6311	94.5571	113.9543	31.3033	0.0000	0.7325	1.7489	3.0978	5.74	5.99
**33**	7.84	-0.1784	1.7688	4.1851	0.9096	12.3918	21.0108	24.7905	0.0000	0.4918	0.6669	3.8289	7.03	7.84
**34**	7.38	-0.1771	1.8347	5.5075	0.8898	18.8846	27.5036	24.7905	18.4396	0.5462	0.7210	4.6236	7.69	7.38
**35**	5.18	-0.1711	2.0002	11.2749	0.7766	56.3799	64.9989	24.7905	0.0000	0.4884	0.5347	4.5949	5.71	5.18
**36**	5.63	-0.1816	1.8802	5.3249	0.6909	67.8246	76.4436	35.9811	0.0000	0.7747	1.0942	3.1464	6.32	5.63
**37**	6.13	-0.1837	1.9154	6.6928	0.7176	70.3284	78.9474	51.4344	0.0000	0.4344	1.0296	2.4707	6.13	6.13
**38**	5.48	-0.1614	1.9124	4.3663	0.7344	61.2523	69.8713	49.4828	0.0000	0.5495	1.0985	1.9388	6.30	5.48
**39**	6.60	-0.1831	1.8388	6.0013	0.7941	45.0084	53.6274	55.9254	18.4396	0.1450	0.6994	1.1946	6.65	6.60
**49**	6.07	-0.2115	1.6141	35.4957	0.9073	0.6369	17.3749	11.1906	0.0000	0.6643	0.6669	2.6865	6.54	6.07
**50**	6.60	-0.1953	1.7365	5.9932	0.9028	12.2549	20.8739	11.1906	0.0000	0.4957	0.4490	3.3765	6.37	6.60
**51**	6.30	-0.1941	1.8015	5.9776	0.7763	43.2575	51.8765	11.1906	18.4396	0.4714	0.3470	3.4573	6.19	6.30
**52**	5.42	-0.1915	1.8800	6.6689	0.7657	49.7503	58.3693	11.1906	0.0000	0.4878	0.3623	3.2201	5.27	5.42
**53**	5.36	-0.1771	1.9122	6.5426	0.7812	49.7503	58.3693	11.1906	0.0000	0.4104	0.6361	3.7671	5.65	5.36
**54**	5.52	-0.1798	1.8774	6.5769	0.8271	37.4954	46.1144	11.1906	0.0000	0.4166	0.6571	3.6761	5.94	5.52

Abbreviation *BLOSUM62*: BLOcks SUbstitution Matrices; *RMSD*: Root-mean-square deviation

Denotation of the scoring functions for MOE molecular docking calculations

S: the final score, which is the score of the last stage of refinement; *E_conf*: the energy of the conformer - if there is a refinement stage, this is the energy calculated at the end of the refinement; *E_place*: score from the placement stage; *E_score1/2*: score from rescoring stages 1 and 2; *E_refine*: score from the refinement stage, calculated to be the sum of the van der Waals electrostatics and solvation energies, under the Generalized Born solvation model (GB/VI)

Denotation of QSAR molecular descriptors

GCUT_SMR_0: the GCUT descriptors using atomic contribution to molar refractivity; *E*: value of the potential energy; *dipoleY*: the y component of the dipole moment (external coordinates); *dipoleZ*: the z component of the dipole moment; *DCASA*: absolute value of the difference between CASA+ (Positive charge weighted surface area, ASA+ times max { qi > 0 }) and CASA- (Negative charge weighted surface area, ASA- times max { qi < 0 }); *Q_RPC-*: relative negative partial charge; *Q_VSA_FHYD*: fractional hydrophobic van der Waals surface area; *SlogP_VSA4*: sum of vi such that Li is in (0.1,0.15]; *vsurf_EDmin1*: lowest hydrophobic energy; *vsurf_IW5*: hydrophilic integy moment; *Q_VSA_PNEG*: fractional negative van der Waals surface area; *Q_VSA_POL*: total positive van der Waals surface area; *SlogP_VSA3*: sum of vi such that Li is in (0,0.1]; *vsurf_ID7*: hydrophobic integy moment; *vsa_other*: approximation to the sum of VDW surface areas (Å2) of atoms typed as "other"; *E_tor*: torsion (proper and improper) potential energy; *balabanJ*: Balaban's connectivity topological index; *vsurf_ID1*: hydrophobic integy moment; *GCUT_SMR_1*: the GCUT descriptors using atomic contribution to molar refractivity.
